# Exploring calibration-free luminescence thermometry with Mn^4+^ anti-Stokes/Stokes emission

**DOI:** 10.1126/sciadv.aeh4432

**Published:** 2026-10-16

**Authors:** Guitong Zhang, JiaPeng Wu, Yixuan Ma, Lizhi Cui, Zhijie Dong, Yuhua Wang, Andries Meijerink

**Affiliations:** ^1^ National and Local Joint Engineering Laboratory for Optical Conversion Materials and Technology of National Development and Reform Commission, School of Materials and Energy, Lanzhou University, Lanzhou, 730000, China.; ^2^ Condensed Matter and Interfaces, Debye Institute for Nanomaterials Science, Utrecht University, Princetonplein 1, 3584CC Utrecht, Netherlands.

## Abstract

Mn^4+^-activated phosphors offer a unique possibility of calibration-free primary luminescence thermometry using the ratio of anti-Stokes to Stokes (AS/S) vibronic emission lines of the ^2^E → ^4^A_2_ transition. Previous work used the AS/S emission of Mn^4+^ for thermometry but always relying on fitting procedures with effective vibrational energies that deviate from actual energies. Here, we explore a strategy without prior temperature calibration using two well-known Mn^4+^-doped hosts, Mg_14_Ge_5_O_24_ (MGO) and K_2_SiF_6_ (KSF). In both Mn^4+^ occupies octahedral sites, replacing Ge^4+^ or Si^4+^, resulting in a weak zero-phonon line (ZPL) and strong vibronic Stokes and anti-Stokes lines. From the temperature-dependent emission spectra the AS/S ratios ν_6_′/ν_6_, ν_4_′/ν_4_, ν_3_′/ν_3_ were determined to serve as input for luminescence intensity ratio (LIR) analysis. For the well-resolved vibronic lines of Mn^4+^ in KSF it is feasible to realize temperature readout without temperature calibration – a capability rarely achieved in luminescence thermometry. In contrast, for Mn^4+^ in MGO vibronic lines exhibit significant overlap, complicating single-phonon-mode modeling. Even spectral deconvolution methods do not allow for reliable calibration-free temperature sensing. This work establishes a generalizable approach to optical thermometry relying on anti-Stokes/Stokes vibronic lines, offering a path towards calibration-free temperature sensing.

## INTRODUCTION

Temperature is an important parameter in science and society (*1*–*3*). In science, its significance spans many disciplines including biology, physics, astronomy and chemistry (*4*–*8*). As the demand for thermometers with improved response time, sensitivity and spatial resolution increases, remote temperature detection utilizing luminescent materials has emerged as a promising avenue (*9*–*11*). Luminescence characteristics such as peak intensity, width and wavelength or luminescence decay time often change with temperature and can be used for temperature sensing. The most popular and accurate methods use luminescence intensity ratios (LIR) to detect temperature (*12*–*15*). The change in intensity ratio of two emission lines with temperature can provide accurate temperature readout that is self-referenced. Especially sharp line emission from two thermally coupled levels of lanthanide ions has been successful. These so-called Boltzmann thermometers can be tuned to achieve optimized accuracy by selecting an energy difference between the two levels that matches the temperature region of interest (*16*). The temperature dependence of the intensity ratio is fitted to the Boltzmann equation with a pre-factor and energy difference Δ*E* as fitting parameters to obtain a calibration curve. A very popular material is NaYF_4_:Er^3+^,Yb^3+^ using green upconversion emission from the thermally coupled ^4^S_3/2_ and ^2^H_11/2_ levels of Er^3+^ after NIR excitation of Yb^3+^. Many other thermally coupled levels of e.g., Nd^3+^, Sm^2+^ or Eu^3+^ have also been successfully used for temperature sensing. In the calibration procedure the fitting parameter Δ*E* gives the energy difference between the thermally coupled levels and the pre-factor is determined by the relative degeneracies of the two levels and the radiative decay rates of the transitions involved for the LIR emission lines. It is interesting that for the same material very different calibration parameters have been reported. For example for NaYF_4_:Er^3+^ values reported for the ^4^S_3/2_ and ^2^H_11/2_ energy difference Δ*E* determined from temperature dependent LIRs vary between 650 and 800 cm^−1^ (*10*–*13*). The origin is not completely understood but discrepancies may arise from the fact that crystal field splitting causes many more than two thermally coupled levels to be involved. For Er^3+^ especially the splitting of the ^2^H_11/2_ level into as many as 6 different crystal field components can explain the variation in reported Δ*E* values and deserves a more detailed analysis. Additionally, instrumental differences contribute to these variations and illustrates that a more unified approach in spectral correction and calibration procedures is needed in the field of luminescence thermometry.

An alternative for lanthanide ions in the field of luminescence thermometry is the group of 3d transition metal ions. Especially Cr^3+^ has been explored utilizing the thermally couples ^2^E and ^2^T_1_ or ^2^E and ^4^T_2_ levels. (*15*) However, also Mn^4+^-activated deep-red emitting phosphors have emerged as a class of materials for optical thermometry. For Mn^4+^ ions in octahedral coordination, the ^2^E_g_ → ^4^A_2g_ parity forbidden transition is characterized by a weak zero-phonon line (ZPL) and vibronic sidebands arising from coupling with odd-parity vibrations (*17*–*19*). The intensity of vibronic side bands is determined by the phonon occupation number *n* for both Stokes (∝*n* + 1) and anti-Stokes(∝*n*) vibronic lines (*20*, *21*). This provides a theoretical foundation for utilizing the intensity ratio of anti-Stokes to Stokes emission peak luminescence intensity ratio (LIR) for temperature sensing (*22*, *23*). Interestingly, for a specific vibrational energy *E*_vib_ the ratio AS/S can be calculated for any temperature without fitting parameters and is determined by the Boltzmann factor $\left(e^{-E_{\text{vib}}/\mathrm{kT}}\right)$ and a pre-factor $\left(\frac{\nu_{\text{AS}}^{3}}{\nu_{S}^{3}}\right)$ to correct for the small difference in density of states (and thus radiative transition rates) at the emission energies for Stokes and anti-Stokes emission. Only the vibrational energy is used as a parameter that can be determined independently of temperature from an emission spectrum. There are many studies on Mn^4+^ doped fluorides and oxides for luminescence temperature sensing relying on the temperature dependence of the decay time, ^2^E-^4^T_2_ emission intensity ratios or anti-Stokes/Stokes intensity ratios (*24*–*26*). Most AS/S studies rely on full-spectrum integration using effective vibrational energies. Attempts to fit single-mode vibrations give fits inconsistent with actual vibrational values (*23*, *27*). However, correction for spectral response of the detection system, spectral conversion to an energy scale with photon flux per constant energy interval and including the density of states pre-factor are typically not considered but are crucial to compare the experimentally measured and theoretically calculated AS/S intensity ratios. In addition, Mn^4+^-doped oxides, while more robust, suffer from strong vibronic sideband overlap which complicates the single-phonon AS/S ratio analysis. These considerations highlight the need for careful analysis of the AS/S intensity for Mn^4+^ to explore the possibility of calibration-free, single-mode AS/S thermometry (*28*–*30*).

It is interesting to note that already for many decades the anti-Stokes/Stokes intensity ratios of vibrational lines in Raman spectra have been used for thermometry, using the same Boltzmann factor. An early and very illustrative paper by Malyj and Griffiths discusses the importance of careful data analysis, including correction for spectral response, conversion of spectra to an energy scale, lamp calibration etc. (*31*). The work also outlines that although no temperature calibration is needed, it is required to calibrate the spectroscopic components such that the true intensities of the AS and S Raman lines can be obtained. The same is true for the calibration-free method presented here: a correct calibration of the spectral response of the measuring system is important. The feasibility of AS/S vibrational Raman thermometry has been demonstrated in many publications, for example in optical fiber temperature sensors, nanoplasmonics and even intracellular temperatures using water (*32*–*34*).

An advantage of AS/S Raman thermometry is that no luminescent probe has to be added. However, Raman signals are notoriously weak and background signals can interfere. The temperature measurements rely on broad spectral features, even tails on the short and long wavelength side of the laser line. Especially in complex systems Raman signals from a range of molecules overlap and temperature calibration is still required. Using the intense AS/S vibronic emission lines from bright luminescent ions gives a better signal/noise ratio and allows for selective/local excitation of micro- or nanocrystalline probes at the position where the temperature needs to be measured. The better signal/noise ratios, narrower spectral features and higher spatial resolution make it worthwhile to explore calibration-free AS/S luminescence thermometry.

In this study, we investigate the performance of Mn^4+^-doped Mg_14_Ge_5_O_24_ (MGO) and K_2_SiF_6_ (KSF) phosphors for temperature sensing and the viability of calibration-free sensing. For MGO, there is significant spectral overlap between broad vibronic peaks while for the KSF phosphor, three distinct vibrational modes (ν_6_, ν_4_, ν_3_) were analyzed to assess the feasibility of achieving a calibration-free AS/S thermometer. The results show that in KSF this is possible when taking into account careful spectral correction and conversion of emission spectra to an energy scale. For ν_6_, ν_4_ good agreement is obtained between measured and calculated AS/S ratios. For Mn^4+^ in MGO vibronic overlap prevents a calibration-free thermometry that cannot be resolved with spectrum deconvolution methods. Nevertheless, MGO exhibits excellent temperature sensing performance up to the highest temperatures possible among oxide hosts, with outstanding thermal cycling stability. These findings confirm the feasibility and accuracy of Mn^4+^-based optical thermometry in both oxide and fluoride matrices and provide evidence for the feasibility of calibration-free luminescence temperature sensing. The absence of the need for temperature calibration makes the system a primary thermometer and this will be discussed, also in comparison to previous reports on luminescent primary thermometers.

## RESULTS

In this section, we first analyze and discuss the structure, optical properties, and temperature-dependent luminescence of Mn^4+^ in Mg_14_Ge_5_O_24_:Mn^4+^ and K_2_SiF_6_:Mn^4+^. Subsequently, we investigate the possibility for calibration-free temperature sensing using the theoretical anti-Stokes/Stokes vibronic intensity ratio and show that for the narrow well-resolved vibronic lines in K_2_SiF_6_:Mn^4+^ experiment and theory are in good agreement providing a sensitive thermometer not requiring prior calibration procedures.

### Room temperature luminescence

To get insight in the luminescence properties of Mn^4+^ in MGO and KSF, excitation and emission spectra were recorded at room temperature. The energy level scheme of Mn^4+^ is shown in Fig. 1A and helps to understand the spectral features observed in the Mn^4+^ luminescence spectra of MGO (Fig. 1B) and KSF (Fig. 1C). In both host lattices Mn^4+^ shows two distinct bands in the excitation spectra corresponding to spin-allowed transitions: ^4^A_2g_ → ^4^T_1g_ transition between 250–350 nm (MGO) and 300–390 nm (KSF), and ^4^A_2g_ → ^4^T_2g_ transition 370–470 nm (MGO) and 400–500 nm (KSF). The emission spectra display the characteristic sharp vibronic lines of six-coordinated Mn^4+^ (*35*). For MGO, four vibronic emission peaks are observed approximately at 624.5, 631.7, 651.7, and 658.7 nm and a weak line at 641.2 nm that is identified as the zero-phonon line (ZPL). The six coordination of Ge^4+^ (and thus Mn^4+^) in MGO is close to octahedral but has slightly deviating bond lengths, while inversion symmetry is maintained (*36*, *37*). For KSF:0.1%, six sharp peaks appear at 597.8, 608.5, 613.0, 630.5, 634.8 and 647.2 nm. The latter peak has a shoulder on the long wavelength side. The emission lines are assigned to Stokes and anti-Stokes vibronic lines of the ^2^E → ^4^A_2_ transition. A weak ZPL is observed between the Stokes and anti-Stokes lines for KSF around 622 nm. These spectra are consistent with previously reported spectra in the literature where also further information on the assignment can be found (*18*–*21*, *23*).

**Fig. 1.**
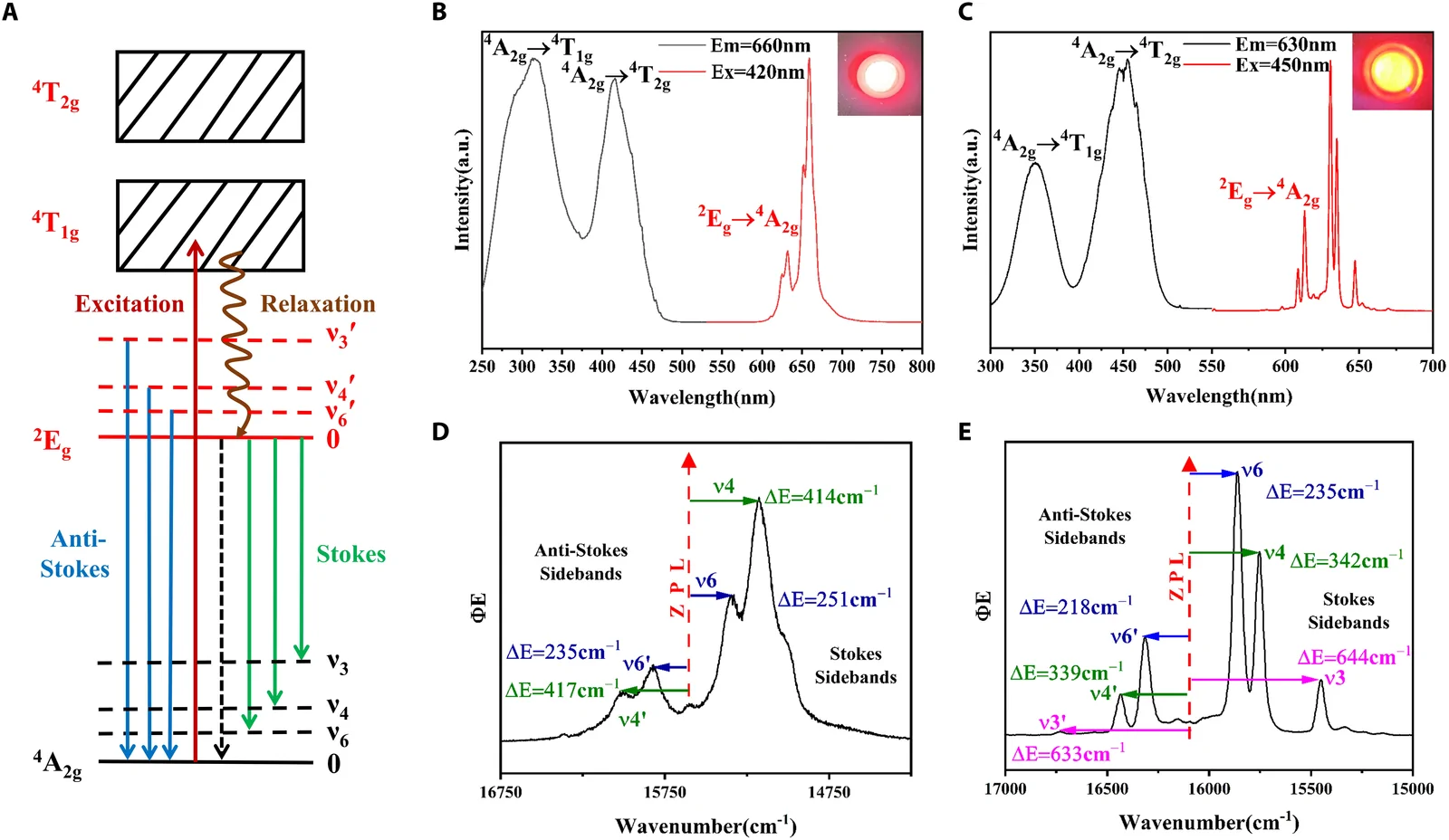
Photoluminescence of Mn^4+^ in MGO and KSF. (**A**) Energy level scheme of Mn^4+^ showing excitation and emission transitions, including Stokes and anti-Stokes vibronics. The black dashed line represents the zero-phonon line which is very weak due to the parity forbidden nature in inversion symmetry. (**B**) Excitation (λ_em_ = 660 nm) and emission (λ_exc_ = 425 nm) spectra of Mg_14_Ge_5_O_24_:1%Mn^4+^. (**C**) Excitation (λ_em_ = 630 nm) and emission (λ_exc_ = 450 nm) spectra of K_2_SiF_6_:0.1%Mn^4+^. Spectra were recorded at room temperature. (**D** and **E**) Higher resolution emission spectra for the ^2^E-^4^A_2_ transition showing a weak zero-phonon line (ZPL) and broad vibronic sidebands for MGO (D) and narrower vibronic emission lines for KSF (E). The vibronic lines are assigned to ν_6_, ν_4_, ν_3_ (Stokes) and ν_6_’, ν_4_’ and ν_3_’ (anti-Stokes) vibronic lines.

The ^2^E → ^4^A_2_ transition is spin and parity forbidden. In octahedral symmetry (and in inversion symmetry in general) the parity selection rule is strict and no/very weak ZPL emission is observed as there are no odd-parity crystal field components to mix in opposite parity states to partially lift the parity selection rule. Coupling with odd-parity vibrations induces odd-parity crystal field components and giving Stokes vibronic lines for the ^2^E_g_ → ^4^A_2g_ transition on the long wavelength side of the ZPL and, at elevated temperatures, anti-Stokes lines appear on the short wavelength side. For both KSF and MGO three odd-parity vibrational modes are typically identified: ν_6_, ν_4_ and ν_3_ (*38*, *39*). In Fig. 1, D and E the Stokes and anti-Stokes vibronic lines are labelled ν_6_, ν_4_, ν_3_ (Stokes) and ν_6_’, ν_4_’ and ν_3_’ (anti-Stokes) and the energy differences with the ZPL are indicated (251 cm^−1^/235 cm^−1^ for ν_6_, 414 cm^−1^/417 cm^−1^ for ν_4_ in MGO and 235 cm^−1^/218 cm^−1^ for ν_6_, 342 cm^−1^/339 cm^−1^ for ν_4_ and 644 cm^−1^/633 cm^−1^ for ν_3_ in KSF). For a proper evaluation of the AS/S intensity ratio it is important that the spectra are corrected for the instrumental response and plotted on an energy scale with photon flux per constant energy interval on the *y*-axis (*40*). The spectra reported here are corrected for the instrumental response after background subtraction and the photon flux per constant wavelength interval (as is recorded during the measurement) is converted to a photon flux per constant energy interval ΦE by multiplication with λ^2^.

### Temperature dependence anti-stokes/stokes vibronics

The theoretical temperature dependence of anti-Stokes/Stokes vibronics is well known (*20*)*.* The intensity of anti-Stokes vibronics is proportional to the phonon occupation number *n* while Stokes vibronics scale with *n* + 1 (*20*). Assuming that the vibration in the ground state and excited state give rise to the same symmetry distortion that allows for admixing of the opposite parity states, the transition probabilities are the same and the intensity ratio is determined by *n*/(*n* + 1) (*14*)

$$n=\frac{1}{e^{\frac{E_{\text{vib}}}{k.T}}-1}$$ (1)

$$\mathrm{LIR}=\frac{I_{\text{AS}}}{I_{S}}=B.\frac{n}{n+1}=B.e^{\frac{-E_{\text{vib}}}{k.T}}$$ (2)

$$B={\left(\frac{\nu_{\mathrm{AS}}}{\nu_{S}}\right)}^{3}$$ (3)

where *E*_vib_ is the energy of the vibration involved, *I* represents the emission intensity, and ν is the frequency of the emission. The factor *B* is a small correction that accounts for the ν^3^ dependence of transition probabilities. The *LIR* of the anti-Stokes and Stokes emission is thus described by the Boltzmann equation and can be written as

$$\text{ln}\left(\mathrm{LIR}\right)=\text{ln}\;\left(\frac{I_{\text{AS}}}{I_{S}}\right)=\text{ln}B-\frac{E_{\text{vib}}}{k.T}$$ (4)

For a vibration of specific energy *E*_vib_ the ratio of the anti-Stokes to Stokes vibronic intensities can be used to determine the temperature without any fitting parameters. This is different from the *LIR* of thermally coupled energy levels of lanthanides where a pre-factor depends on the radiative transition probabilities to the final levels which influences the intensity ratio while also crystal field splitting complicates the analysis. For the calibration-free temperature analysis it is important that spectra are corrected for the instrumental response and plotted as photon flux per constant energy interval ΦE vs. energy as outlined above.

### Temperature dependent luminescence MGO:Mn^4+^

To investigate the viability of using the *I*_AS_/*I*_S_ intensity ratio for temperature sensing, temperature dependent emission spectra were recorded of MGO:Mn^4+^ with 20 K intervals between ∼100 and 500 K (Fig. 2). MGO:Mn^4+^ is a well-known deep red emitting phosphor where the Mn^4+^ luminescence quenching temperature is very high. The overview of spectra in Fig. 2A shows the trends: the relative intensity of the anti-Stokes vibronics increases with temperature (*28*). while all vibronic lines broaden. The width of the vibronics lines is broader than for KSF:Mn^4+^, already at low temperatures (vide infra). The broadening may result from the lower symmetry of the MGO structure, including the lower than octahedral symmetry for the Ge^4+^/Mn^4+^ with different bond lengths (4 + 2) to the six coordinating ligands while also some mixed occupation of the Ge^4+^- Mg^2+^ sites may contribute to inhomogeneous broadening. Examples of spectra at 257, 357 and 477 K are shown in Fig. 2 (B to D). The emission peaks redshift upon heating from 97 K to 477 K with a total shift of ∼92 cm^−1^. The shift can be explained by a crystal expansion which affects the octahedral field environment of Mn^4+^ slightly and results in a small redshift, as is typically observed for the ^2^E-^4^A_2_ emission of Mn^4+^ and also Cr^3+^. The shift will not affect the temperature measurement relying on the anti-Stokes to Stokes intensity ratio. The observations are in line with earlier work on MGO:Mn^4+^.

**Fig. 2.**
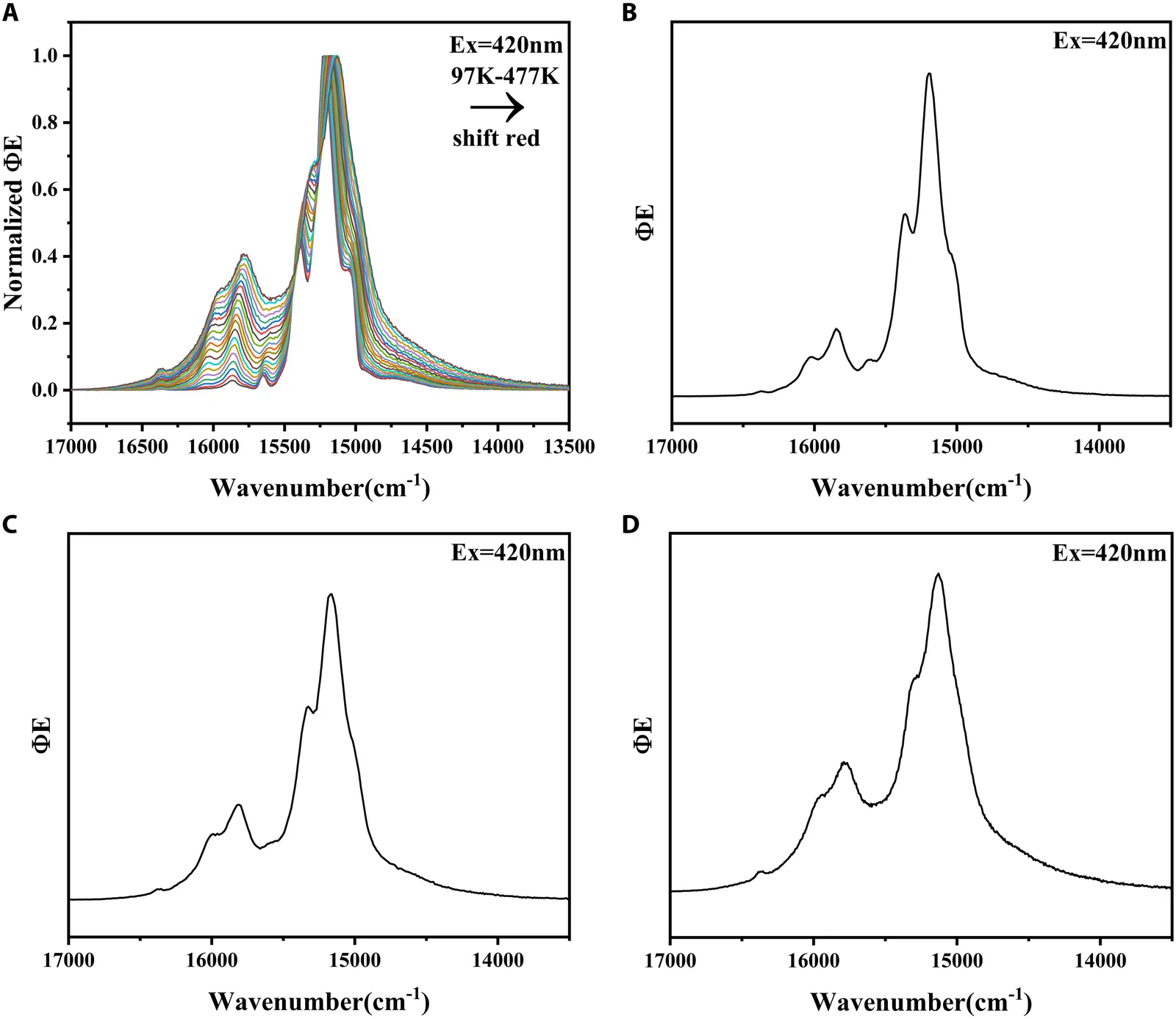
Temperature dependent Mn^4+^ luminescence in MGO. (**A**) Normalized temperature-dependent emission spectra of Mg_14_Ge_5_O_24_:1% Mn^4+^ under the 420 nm excitation (97 K–477 K). (**B** to **D**) Selection of emission spectra of the Mg_14_Ge_5_O_24_:1% Mn^4+^ at different temperatures, viz. 257 K, 357 K and 477 K.

The *LIR I*_AS_/*I*_S_ was determined for the two stronger vibronic lines ν_6_ and ν_4_ by determining the ratio of the intensities for the peak maxima: ν_6_′/ν_6_ and ν_4_′/ν_4_. In Fig. 3, A and B the ratios at different temperatures are plotted as ln(*LIR*) vs. 1/*T* together with the a line for ln(*I*_AS_/*I*_S_) vs. 1/*T* calculated using Eq. (4). For the calculations an average energy *E*_vib_ of 243 cm^−1^ was used for ν_6_ and 415.5 cm^−1^ for ν_4_ as determined from the emission spectra in Fig. 1D. From the energies of the peak maxima of the anti-Stokes and Stokes vibronic lines the value for *B* is calculated using Eq. (3) to be 1.098 for ν_6_ and 1.173 for ν_4_. There is a clear mismatch between the theory and experiment. Fitting the experimental results to a Boltzmann equation gives a good fit for ν_6_’/ν_6_ with *E*_vib_ = 196 cm^−1^ and *B* = 1.081 (Fig. 3C) and for ν_4_’/ν_4_ with *E*_vib_ = 369 cm^−1^ and *B* = 0.95 (Fig. 3D). These values deviate from the theoretically expected values and show that calibration-free temperature sensing using peak intensities is not reliable for MGO:Mn^4+^.

**Fig. 3.**
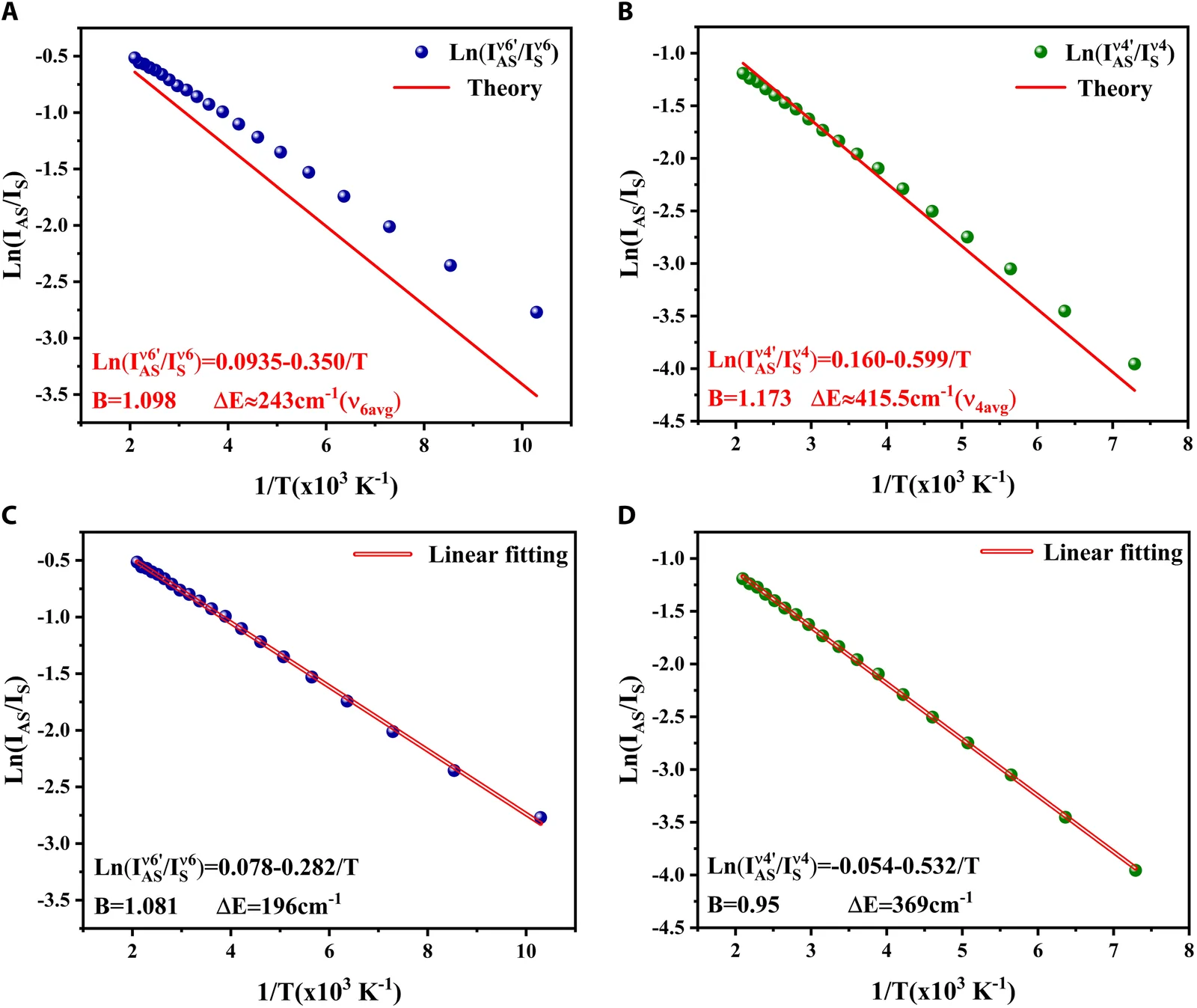
Temperature sensing performance of MGO:Mn^4+^. Comparison between experimentally measured and theoretical intensity ratio of anti-Stokes/Stokes vibronics for (**A**) ν_6_ and (**B**) ν_4_. The red lines present calibration-free calculations of the intensity ratio using Eq. (4) and a vibrational energy of 243 cm^−1^ for ν_6_ and 415.5 cm^−1^ for ν_4_ as determined from Fig. 1D. (**C** and **D**) Same data points with the red lines showing fits to Eq. 4. Fitting parameters for the best fit are indicated.

The anti-Stokes/Stokes *LIR* of Mn^4+^ vibronics has been used before for temperature sensing (*15*, *23*). For MGO:Mn^4+^ a good fit for ln(*I*_AS_/*I*_S_) vs. 1/T was obtained with a slope *E*_vib_/k = 479 K corresponding to E_vib_ = 332 cm^−1^, a value in between the energies of ν_4_ and ν_6_. This is not unexpected as integration included all vibronic lines. Also, intensities seem to have been obtained from emission spectra on a wavelength scale. The same approach was used for temperature sensing with Mn^4+^ AS/S vibronics in Y_2_MgTiO_6_, again integrating over the full vibronic spectrum. Using AS/S intensity ratios for specific vibrational modes has been rarely explored. An example is KSF:Mn^4+^ where the AS/S vibronic intensities of ν_6_ were analyzed and the fit result gave ln*B* = 0.215 (*B* = 1.24) and E_vib_/k = 363 K (E_vib_ = 252 cm^−1^) which deviates from theoretical values (vide infra). Clearly, so far no successful calibration-free temperature sensing using Mn^4+^ vibronics has been reported.

The difference between experiment and theory can be caused by the strong overlap between the broad vibronic lines which impacts the intensity of each emission peak by other nearby vibronic emission peaks. A possible solution to this is to determine intensities of individual vibronic lines by deconvolution methods and fitting the emission spectra to a sum of Gaussian or Lorentzian lines. Both a sum of six Gaussians or Lorentzians gives a close agreement with experimental emission spectra (Fig. 4, A and B and figs. S7 and S8). The temperature dependence of the intensity ratios *I*_AS_/*I*_S_ however still strongly deviates from the theoretical dependence (data points vs. red lines in Fig. 4, C and E). A good fit to Eq. 4 can be obtained but again with vibrational energies that are significantly lower than the experimentally determined vibrational energies (Fig. 4, D and F). One may speculate about the reason of the deviations in Fig. 4, C and E. Possibly, the Gaussian (or Lorentzian, see figs. S7 and S8) fits are not capable of correctly extracting intensities of individual vibronic AS and S lines. Alternatively, the broad vibronic lines may contain contributions of multiple vibrations resulting in effective vibrational energies that are lower than what is determined from the peak maxima. In any case, it is evident that for MGO:Mn^4+^ with broad and overlapping vibronic anti-Stokes and Stokes lines calibration-free temperature sensing is not possible.

**Fig. 4.**
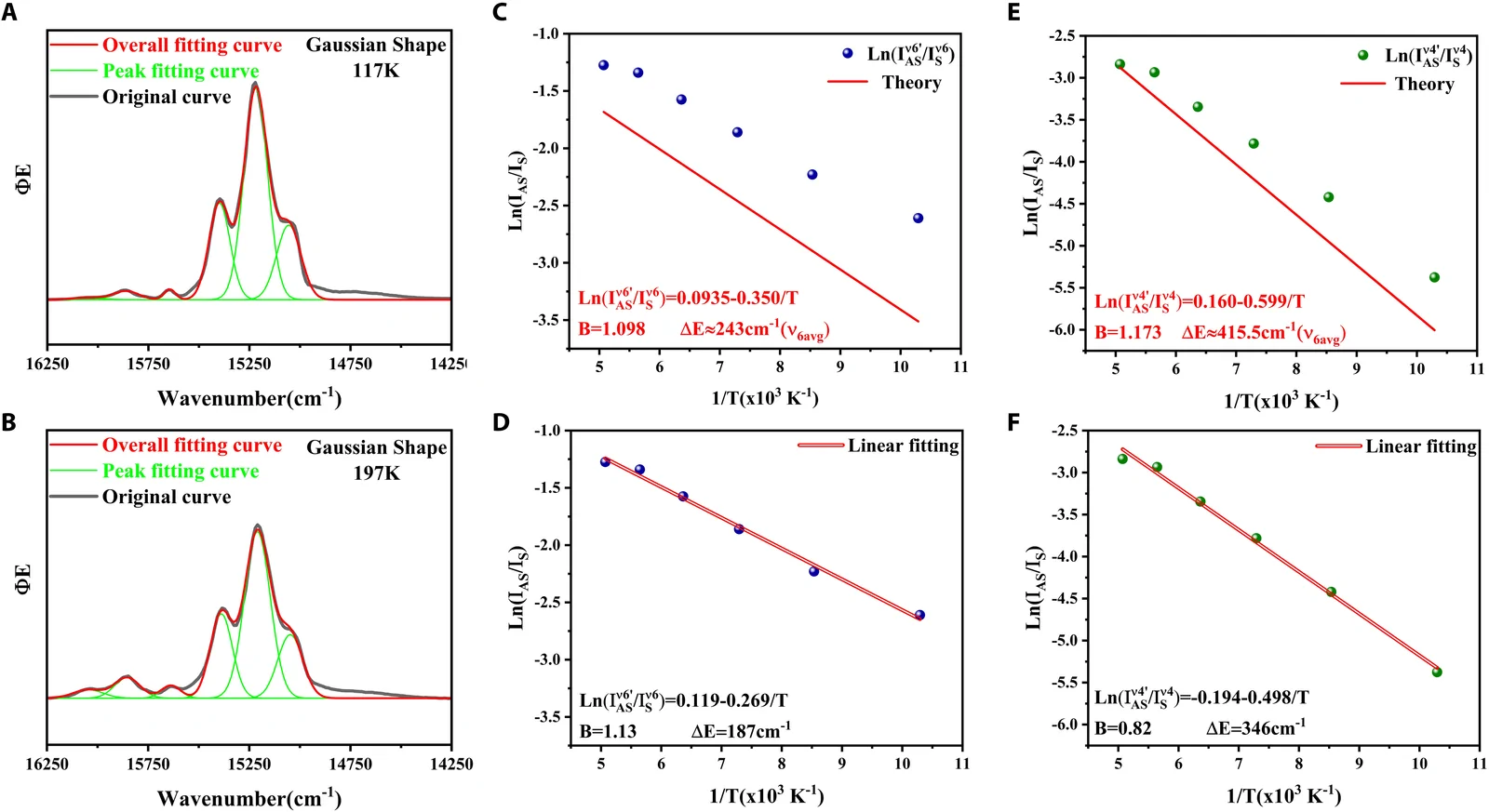
Exploring calibration-free temperature sensing in MGO:Mn^4+^. (**A** and **B**) Examples of emission spectra of MGO:Mn^4+^ at two temperatures fitted to a sum of six Gaussians to extract the intensities of individual Stokes and anti-Stokes vibronic lines. In (**C**) the temperature dependence of the *LIR* for ν_6_’/ν_6_ and (E) for ν_4_’/ν_4_ are plotted together with the theoretical temperature dependence for intensity ratios determined from the Gaussian fits. In (**D**) and (**F**) fits to Eq. 4 are depicted for the same data points. Fitting parameters are included in the Figures.

In spite of the failure to realize calibration-free temperature sensing for MGO:Mn^4+^, the excellent fits to Boltzmann Eq. 4 can be used for accurate temperature sensing. Similar to luminescence thermometers using the LIR of thermally coupled energy levels, the absolute sensitivity and relative sensitivity can be calculated by the following equations (*41*)

$$S_{A}=\frac{d\left(\mathrm{LIR}\right)}{\mathrm{dT}}=B.\text{exp}\left[\frac{-\mathrm{\Delta E}}{\mathrm{kT}}\right].\left[\frac{\mathrm{\Delta E}}{kT^{2}}\right]$$ (5)

$$S_{R}=\frac{1}{\mathrm{LIR}}\frac{d\left(\mathrm{LIR}\right)}{\mathrm{dT}}=\left[\frac{\mathrm{\Delta E}}{kT^{2}}\right]$$ (6)

The temperature dependence of *S*_R_ is shown in Fig. 5 (A and B). High values of *S*_R_ at low temperatures quickly drop when the change in *n*/(*n* + 1) decreases for higher phonon occupation numbers *n*. However, even though *S*_R_ is high at low temperatures the very weak intensity of the AS vibronic lines reduces the signal-to-noise ratio. The optimum temperature range is determined by *E*_vib_ and in analogy with the thermally coupled levels the optimum range is for *E*_vib_/kT = 2–3.41 (*10*). In Fig. 5 (C and D) temperature cycling experiments are show high stability and reproducibility for MGO:Mn^4+^ as luminescent thermometer. In the Supporting Information table S1 further analysis of these data is included, showing that temperature errors below 1 K are realized in the low temperature regime.

**Fig. 5.**
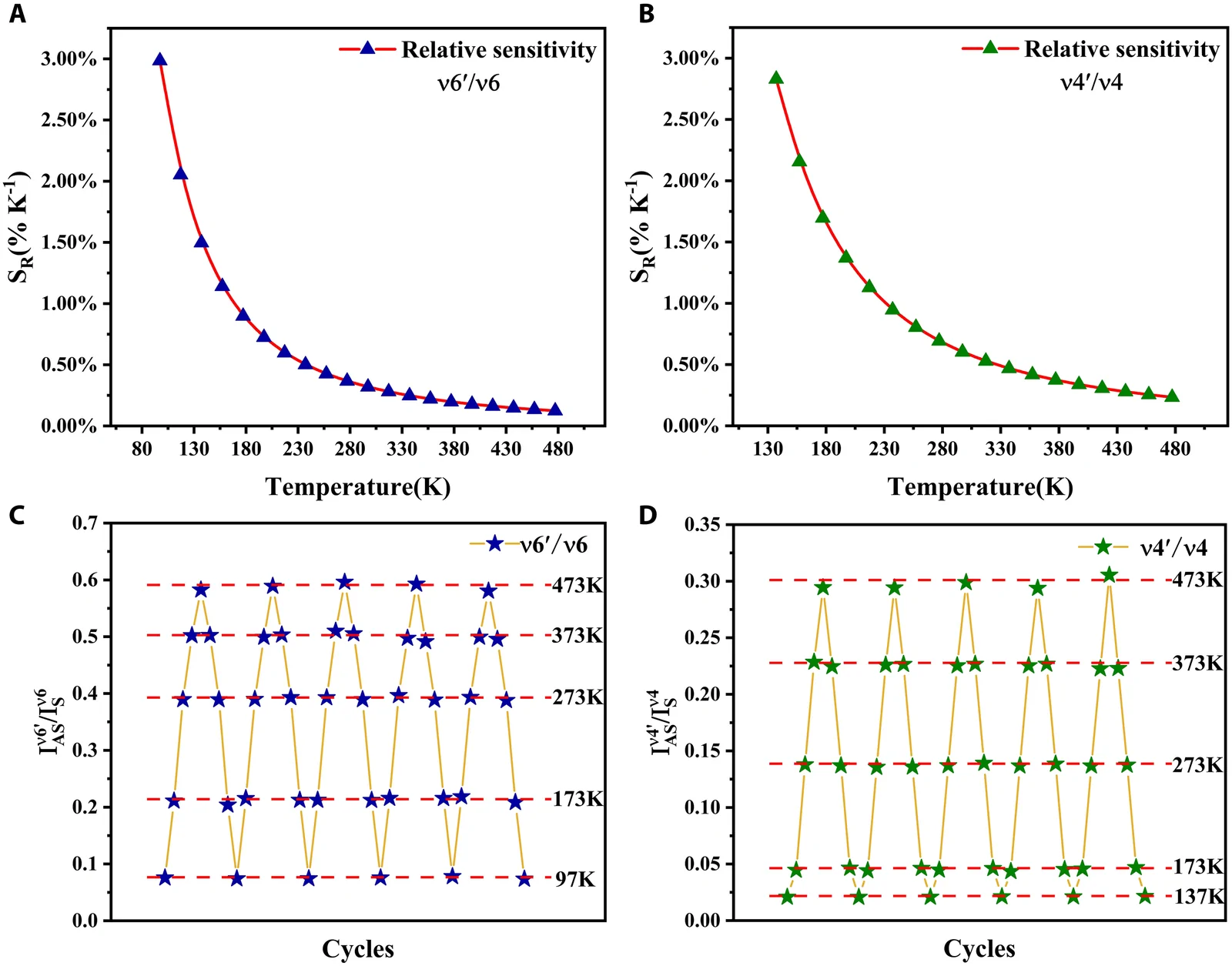
Temperature sensitivity and thermal cycling of MGO:1%Mn^4+^. (**A** and **B**) Relative sensitivity *S*_R_ values for the AS/S vibronic intensity for the ν_6_ and ν_4_ vibrational modes in MGO:1%Mn^4+^ according to the fits Fig. 3 (C and D). Similar results can also be obtained for ν_3_ and the total AS/S vibronic intensity ratios (see Supporting Information fig. S5). (**C** and **D**) Thermal cycling experiments showing the stability and reproducibility of temperature sensing relying on the AS/S vibronic intensity ratio for MGO:Mn^4+^ using the ν_6_ (C) or ν_4_ (D) vibronic lines.

### Temperature dependent luminescence KSF:Mn^4+^

The most widely studied Mn^4+^-doped phosphor is without doubt K_2_SiF_6_:Mn^4+^. The efficient narrow line emission around 630 nm, relatively strong absorption around 450 nm and high luminescence quenching temperature make this material a successful red phosphor for application in blue-green-red emitting LEDs as backlights in LCD displays. The long emission lifetime and stability issues make the material less suitable for high power white light LEDs for lighting. Here we explore the narrow line anti-Stokes and Stokes vibronic lines for parameter free luminescence temperature sensing. The much narrower linewidth may prevent the problems encountered for MGO:Mn^4+^ discussed above. Interestingly, so far only a few reports exist and luminescence temperature sensing using KSF:Mn^4+^ and the feasibility of calibration-free luminescence temperature sensing has never been demonstrated.

To investigate the feasibility of calibration-free temperature sensing using the AS/S ratio of the vibronic emission lines, high-resolution variable-temperature spectra were measured at intervals of 10 K from ∼200 K to 500 K. In Fig. 6A, an overview of temperature dependent emission spectra of KSF:0.1%Mn^4+^ is shown. Just as for MGO:Mn^4+^ the relative intensity of the AS emission lines increases with temperature and a small redshift (for MGO ∼80 cm^−1^ and for KSF ∼25 cm^−1^ from 197 K to 477 K). Different from MGO, narrow and well-resolved vibronic lines are observed. There is temperature induced broadening but the overlap is small up to high temperatures. Examples of narrow line emission spectra are shown in Fig. 6 (B to D).

**Fig. 6.**
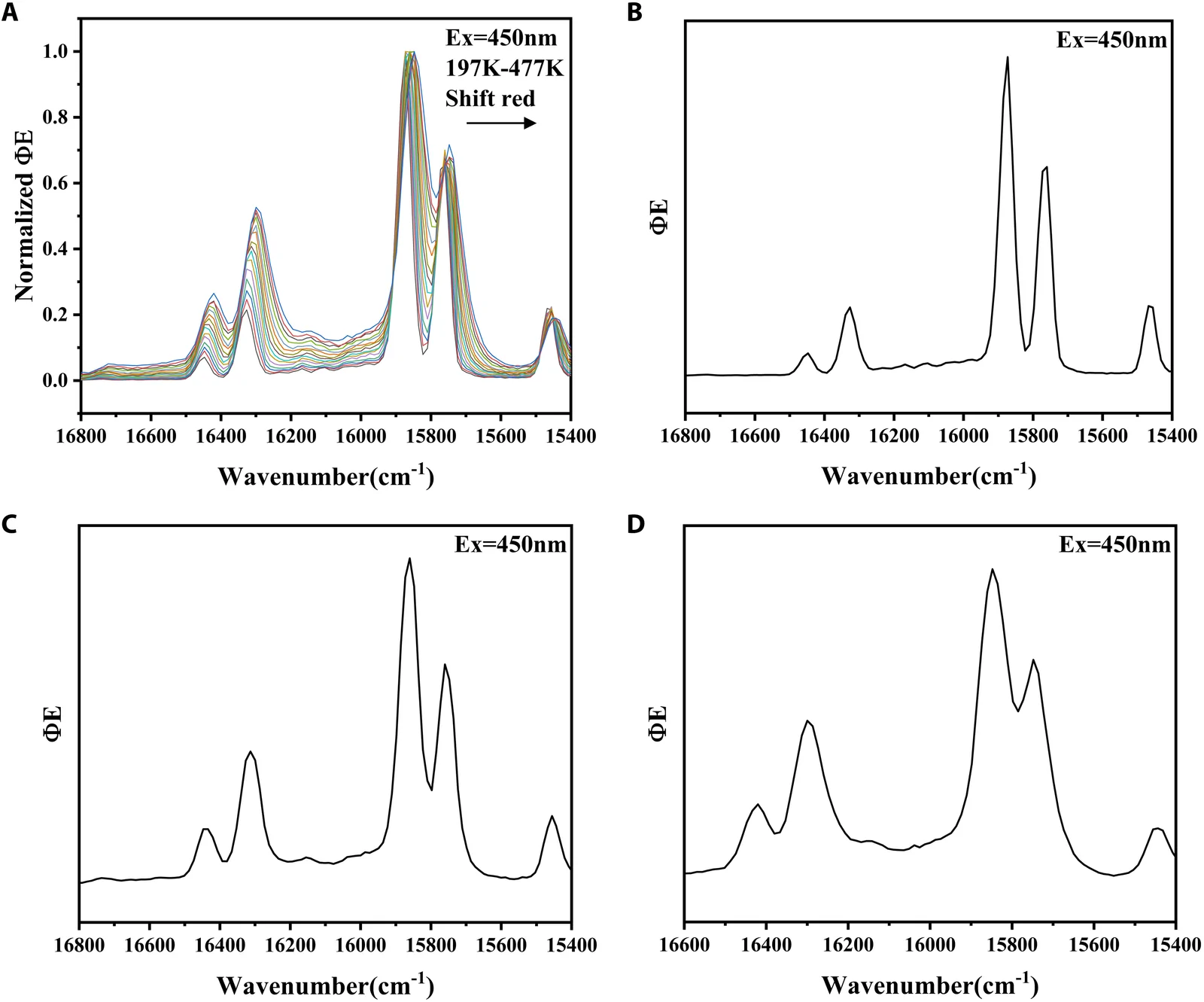
Temperature dependent luminescence of Mn^4+^ in KSF. (**A**) Normalized temperature-dependent emission spectra of KSF:0.1% Mn^4+^ for 450 nm excitation (197 K–477 K), (B to D) Emission spectra of KSF:0.1% Mn^4+^ at 197 K (**B**), 337 K (**C**) and 477 K (**D**).

The AS/S intensity ratio was determined from the peak maxima for all three vibrational modes ν and also for the integrated AS and S vibronic intensities. In Fig. 7 the temperature dependence of ν_6_′/ν_6_, ν_4_′/ν_4_, ν_3_′/ν_3_ and ν_tot_′/ν_tot_ are shown (plotted as ln (*I*_AS_/*I*_S_) vs. 1/*T* where a straight line is expected). The experimentally measured AS/S intensity ratios are plotted together with the theoretically calculated ratios using Eqs. 2 to 4 and the average vibrational energies determined from the room temperature emission spectra in Fig. 1E, viz. ν_6_ = 226.5 cm^−1^ ν_4_ = 340.5 cm^−1^ and ν_3_ = 638.5 cm^−1^. The pre-factor B was calculated from the actual emission energies of the AS and S emission lines in Fig. 1E. Especially for ν_6_′/ν_6_ (Fig. 7A), but also for ν_4_′/ν_4_ (Fig. 7B) there is a good agreement between the theoretically calculated intensity ratio and the experimentally measured intensity ratio. This demonstrates that the AS/S intensity ratio as determined from the corrected emission spectra plotted on an energy scale can be used for calibration-free temperature sensing. The availability of a parameter luminescence temperature sensing reference material is important to directly measure temperature without the need for calibration. It provides a reference for comparison between different set-ups used for luminescence temperature sensing and gives the possibility to test the temperature calibration for different heating devices, for example to check if the temperature indicated is identical to the (surface) temperature of the luminescent material in the heating device. While the agreement between theory and experiment is very good for ν_6_ and ν_4_, there is a clear deviation for ν_3_′/ν_3_ (Fig. 7C). The very low intensities of the AS vibronic lines for the high energy ν_3_ vibration hamper a reliable determination of the intensity ratio. For the total integrated vibronic area the experimentally found ratios closely follow the theoretically expected temperature dependence for a single vibration of energy ν_6_ (Fig. 7D). The observed temperature dependences were also fitted to Eq. 4 and plotted in Fig. 7 (E to H). For ν_6_ the parameters found for *B* and *E*_vib_ are very close to the theoretically calculated values (*E*_vib_ = 214 cm^−1^ vs. 226.5 cm^−1^ and *B* = 1.02 vs. 1.088) confirming the feasibility of calibration-free temperature sensing. For ν_4_ the values show a deviation (*E*_vib_ = 300 cm^−1^ vs. 340.5 cm^−1^ and B = 0.94 vs. 1.135). Possibly, the smaller slope is due to some overlap with the lower energy strong ν_6_ vibronic lines due to peak broadening which lowers the experimentally found *E*_vib_. When doing a fit with a fixed correct value for *B* of 1.135, the value for *E*_vib_ for the best fit is 339 cm^−1^, close to the actual vibrational energy of ν_4_. For the integrated vibronic intensities a good theoretical description is provided using similar parameters as found for ν_6_ (*E*_vib_ = 235 cm^−1^ and *B* = 1.09). Fitting the experimental data gives an excellent fit for *E*_vib_ = 241 cm^−1^ and *B* = 1.11 (Fig. 7H). This shows that the strongest and lowest energy vibronic line ν_6_ dominates the temperature dependence.

**Fig. 7.**
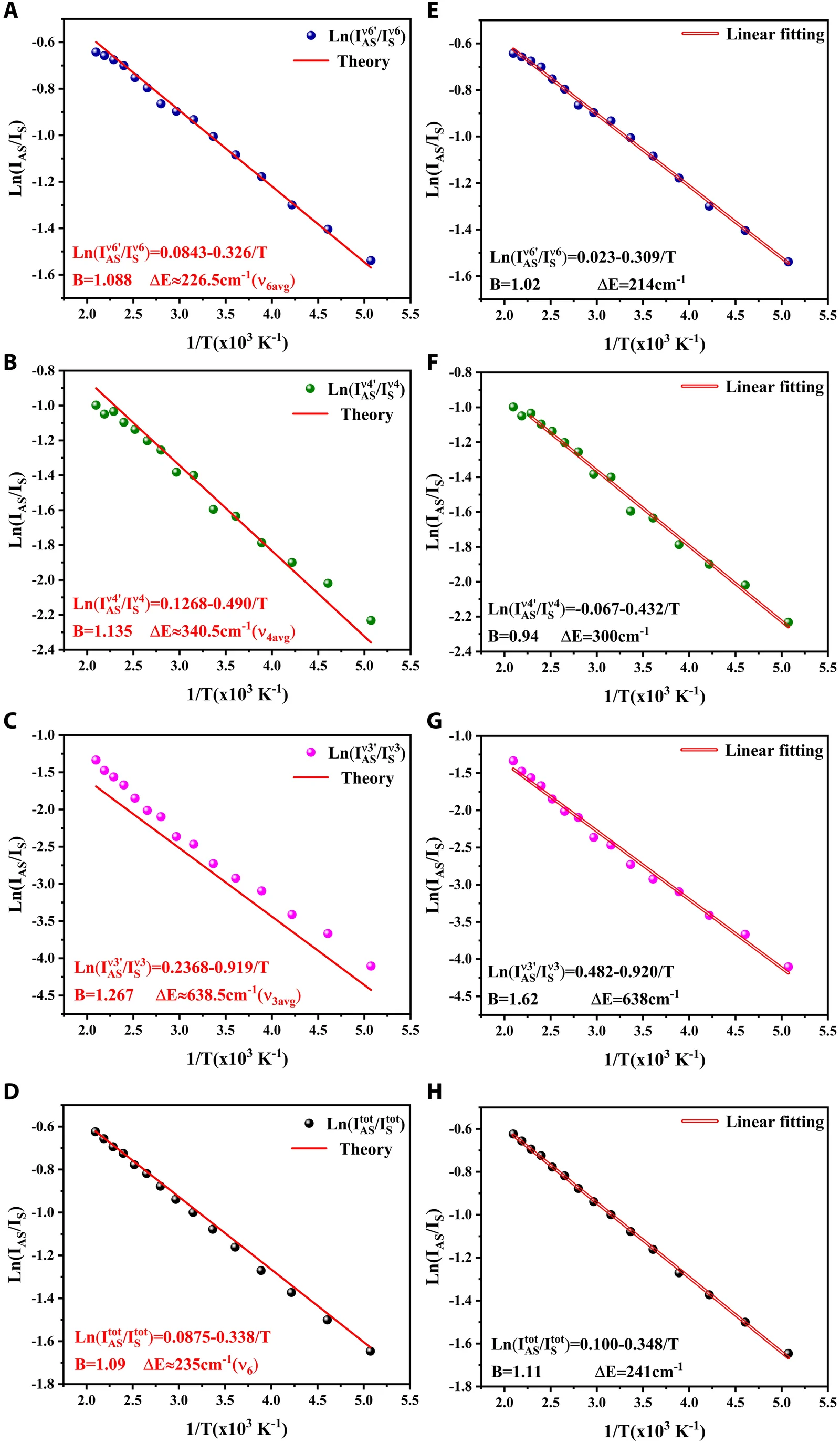
Exploring calibration-free temperature sensing in KSF:Mn^4+^. (**A** to **D**) Emission intensity ratios of *I*_AS_/*I*_S_ for ν_6_/ν_6_′, ν_4_/ν_4_′ and ν_3_/ν_3_′ (Peak Height), ν_tot_/ν_tot_′ (total peak area) vibronic lines in KSF:0.1%Mn determined from the temperature dependent emission spectra in Fig. 6A. Drawn lines in (A to D) are theoretically calculated ratios using Eqs. 2 to 4 and the vibrational energies for ν_6_, ν_4_ and ν_3_ determined from the spectrum in Fig. 1D. (**E** to **H**) Same data points as in (A to D) fitted to Eq. 4. The fitting parameters are included in the figures.

It is interesting to compare the present results to earlier studies where the AS/S vibronic intensities of Mn^4+^ in KSF and related compositions as K_2_TiF_6_:Mn^4+^ (KTF) were investigated for temperature sensing (*23*, *42*). For Mn^4+^ in KTF the logarithm of the integrated AS/S vibronic intensity ratio (as determined from spectra on a wavelength scale) versus 1/*T* showed the expected linear relationship. A good fit was obtained for an effective vibrational energy of 283 cm^−1^, which is in between the energies for the strongest vibronic lines for ν_6_ (216 cm^−1^) and ν_4_ (325 cm^−1^). Reports on luminescence thermometry relying on a single Mn^4+^ vibronic line are rare. Shao *et al.* used the KSF ν_6_ vibronic AS/S ratio relying on peak intensities similar to our procedure. Fitting of ln(*I*_AS_/*I*_S_) vs. 1/T yielded a good fit for a vibrational energy of 252 cm^−1^, a value that deviates from actual vibrational energy of 226 cm^−1^ (*23*). The AS/S intensities were obtained from spectra on a wavelength scale and it is not clear if and how the spectra were corrected for the instrumental response. Also, there was no correction for the ν^3^ dependence of radiative transition probabilities. The present results show that the AS/S ratio for narrow vibronic lines of Mn^4+^ can be used for reliable calibration-free luminescence thermometry if the emission spectra are corrected for the instrumental response and converted to an energy scale.

To evaluate the performance as a luminescence thermometer, the relative sensitivities *S*_R_ were determined from the fits of the temperature dependence [Eq. 4 for ν_6_ (217 cm^−1^), ν_4_ (300 cm^−1^), ν_3_ (629 cm^−1^), ν_tot_ (239 cm^−1^)] as shown in Fig. 7 (E to H). The temperature dependence of *S*_R_ in Fig. 8 shows the typical behavior of a Boltzmann thermometer: *S*_R_ rapidly drops with increasing temperature. For higher vibrational energies *E*_VIB_ the values for *S*_R_ remains high up to higher temperatures. The behavior is very similar to that observed for emission from two thermally coupled levels. Different from two-level Boltzmann thermometers there it is not possible to realize a large pre-factor. Note that using a large pre-factor as a result of a higher radiative decay rate from the higher energy level is beneficial enhances the relative intensity of the weaker emission line. This boosts the signal-to-noise ratio and allows for more precise temperature measurements. This is for example the case for example for the ^2^H_11/2_ and ^4^S_3/2_ thermally coupled levels of Er^3+^ (*10*). The same principle that allows for calibration-free thermometry using anti-Stokes/Stokes LIR (i.e. same transition dipole moment for anti-Stokes and Stokes vibronic lines) thus also has a drawback as it prevents enhancement of the weaker high energy emission by selecting a higher energy emission with a larger oscillator strength.

**Fig. 8.**
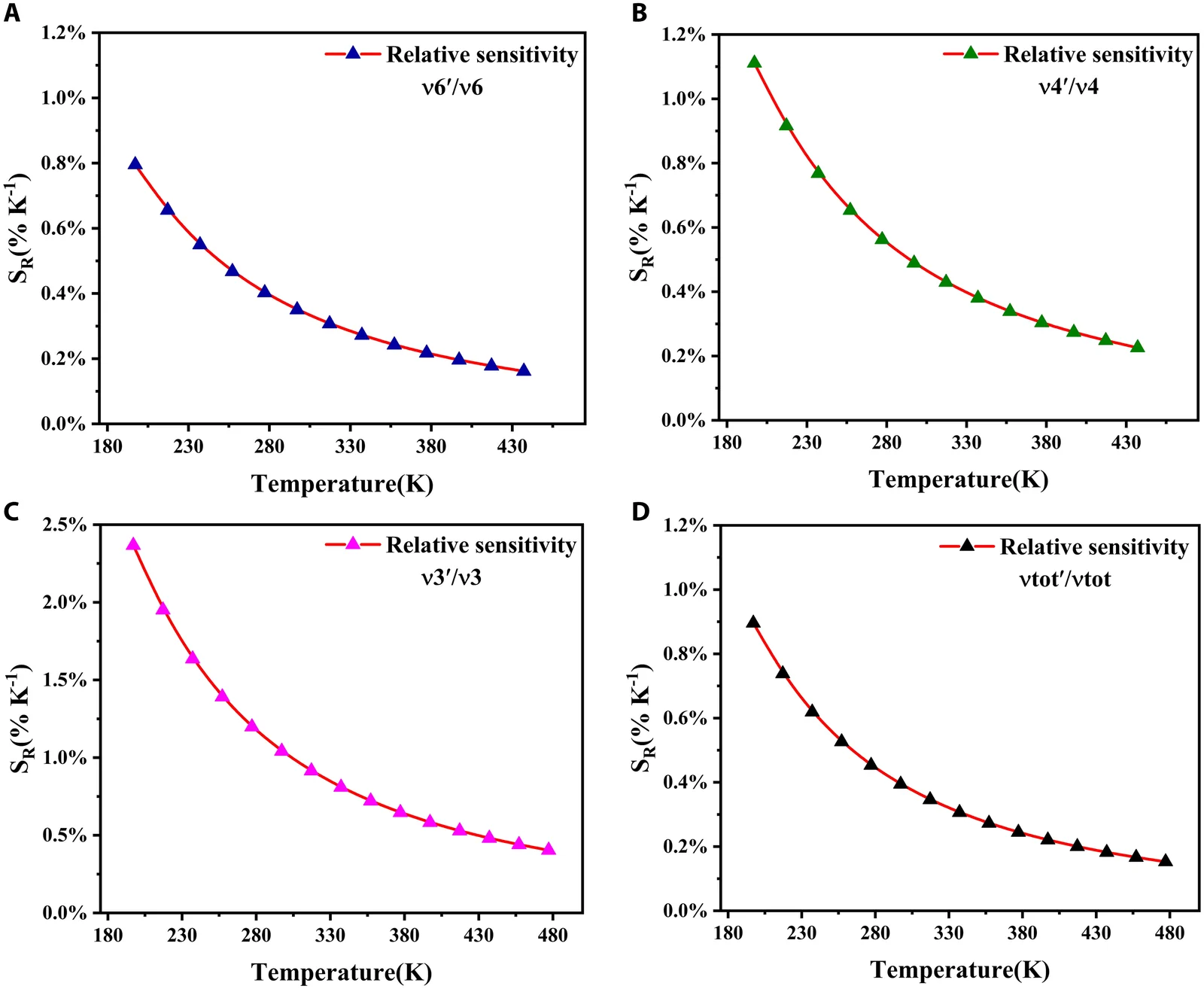
Temperature sensitivity of KSF:0.1%Mn^4+^. (**A** to **D**) The Relative Sensitivity *S*_R_ values for luminescence thermometry relying on the AS/S intensity ratio for the different vibrational modes for (A) ν_6_ (226 cm^−1^), (B) ν_4_ (340 cm^−1^), (C) ν_3_ (629 cm^−1^) and (D) ν_tot_ (239 cm^−1^).

To obtain insight in the accuracy and variation in temperature measurements based theoretical calculation of the temperature from the AS/S LIR of vibronic lines, thermal cycling experiments were performed by heating the KSF:Mn^4+^0.1% material from 197 K to 423 K in five cycles and measuring the temperatures for the cooling and heating stage. The results are shown in Fig. 9. The errors between the setpoint of the heating stage and the calculated temperatures are depicted. The average error varies between 1.5 and 5 K up to 400 K. At higher temperatures the deviations increase. The best performance is around 300 K for the 226 cm^−1^ ν_6_ vibronic lines. The performance is not as good as for optimized Boltzmann LIR thermometers based on careful prior temperature calibration. Further research on the origin of the temperatures deviations is in warranted and possible causes will be discussed in the discussion section. In spite of the deviations it is encouraging to how close the AS/S vibronic intensity ratio predicts the actual temperature based on a single independently measured vibrational energy.

**Fig. 9.**
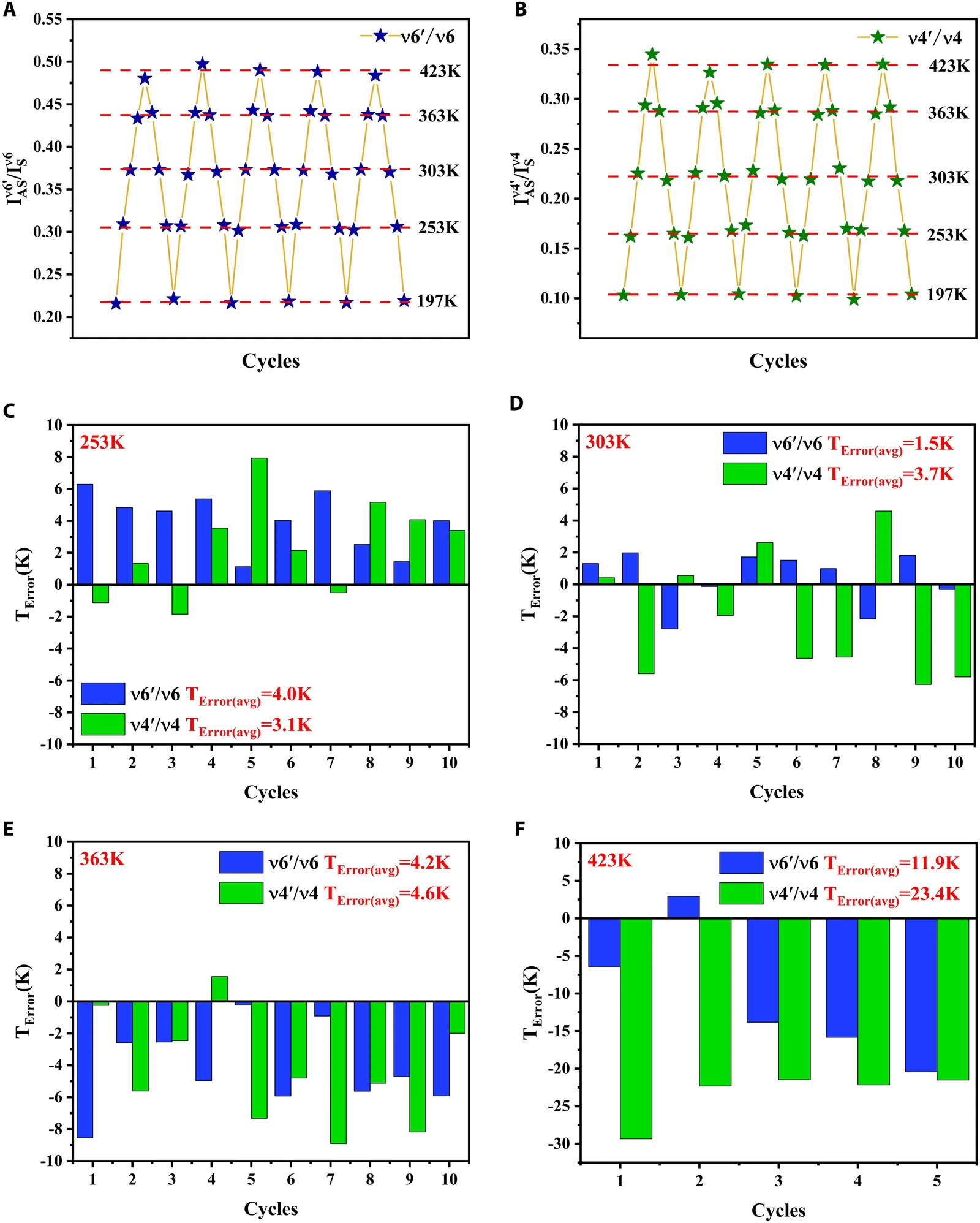
Thermal cycling and error analysis. (**A** and **B**) Thermal cycling experiments showing the accuracy and reproducibility of temperature sensing relying on the AS/S vibronic intensity ratio for KSF:Mn^4+^ using the ν_6_ or ν_4_ vibronic lines. The differences between the calculated temperatures and the setpoint for both the heating and cooling stage for 253 K (**C**), 303 K (**D**), 363 K (**E**) and 423 K (**F**) in the five cycles are plotted. The average deviations are given for both ν_6_ and ν_4_ at each temperature.

## DISCUSSION

The field of luminescence thermometry has seen impressive progress in the past decade and many luminescent (nano)materials have been discovered that allow for accurate temperature sensing by remotely measuring luminescence spectra. So far all systems rely on prior calibration of the temperature dependence of the luminescence. However, calibration can be flawed and often calibration curves reported for the same materials show significant differences in fitting parameters. These variations hamper the reliability and general applicability of luminescence thermometry. A potential calibration free method for luminescence thermometry is using the anti-Stokes/Stokes (AS/S) intensity ratio of vibronic lines. The theoretical intensity ratio can be calculated from the energy of the vibration and temperatures can be directly determined without fitting parameters. So far successful calibration-free luminescence thermometry has not been reported. Here, the ^2^E → ^4^A_2_ emission of Mn^4+^ is explored as it provides an ideal probe with a weak (spin- and parity forbidden) zero-phonon line accompanied by strong Stokes and anti-Stokes vibronic lines. Two model systems with high quenching temperatures for the Mn^4+^ luminescence were investigated: MGO:Mn^4+^ and KSF:Mn^4+^. Emission spectra were measured over a wide temperature range. For a correct evaluation of the luminescence intensity ratios (LIR) spectra were corrected for instrumental response and converted to an energy scale.

Comparison of the experimentally observed and theoretically calculated temperature dependence of the AS/S LIR showed a clear deviation for MGO. This is explained by broad and overlapping vibronic bands which prevents accurate intensity measurements for individual vibronic lines. On the other hand, for the sharp vibronic lines of Mn^4+^ in KSF the experimentally measured LIR for the ν_6_ and ν_4_ vibrations was in good agreement with the theoretically calculated LIR confirming the potential of Mn^4+^ luminescence for parameter free temperature sensing. The present results demonstrate the feasibility of calibration free luminescence thermometry. This is not only important for reliable and accurate remote temperature sensing but also gives the possibility to independently and directly determine the temperature of luminescent materials in heating systems for different experimental configurations.

The ability to measure temperature without prior calibration against a more accurate thermometer constitutes primary thermometers. Based on independently determined physical parameters the temperature can be calculated (*43*). Clearly, KSF:Mn^4+^ qualifies as a primary thermometer: only the vibrational energy needs to be determined (from emission spectra, at any temperature) and the temperature can be calculated from the AS/S intensity ratio (where of course a well-calibrated spectrometer and proper correction of the emission spectra is required to determine the true intensity ratio). In the field of luminescence thermometry a limited number of primary thermometers has been reported (*44*–*46*). In an excellent overview in the book ‘Luminescent Thermometry’ primary luminescent thermometers are reviewed (*44*).

Interestingly, for all different types of primary thermometers described in this book chapter some form of temperature calibration is still required, either by measuring the bandgap E_g_ as a function temperature (for semiconductor nanocrystals) or calibration at a single fixed temperature T_0_ to establish the pre-factor for LIR thermometers. Depending on the definition of primary thermometers, this would make these materials pseudo-primary thermometers. The main advantage of the present AS/S LIR thermometer is that the transition dipole moments for the AS and S vibronic transitions are the same (just as for AS/S Raman thermometry) while for thermally coupled energy levels it is not possible to independently and accurately calculate the ratio of the transition probabilities of the different transitions from the thermally coupled levels, not even with Judd-Ofelt theory. Determination of the pre-factor therefore requires calibration at least at one temperature.

The present performance of the KSF:Mn^4+^ AS/S thermometer has not been optimized in this work. Average deviations from the actual temperatures exceed 1 K and especially in the high temperature range the theoretically calculated temperature deviates from the setpoint of the heating stage. This may be partly related to the actual temperature at the surface of the microcrystalline powder layer being lower than the setpoint due to thermal insulation of the layer. We are presently investigating the role of layer thickness and find this plays a role. The spectra in the thermal cycling series were recorded using a scanning monochromator and this may lead to small intensity changes during the scan that affect the AS/S ratio. Future experiments with continuous detection using a CCD camera are warranted to better understand the reproducibility. Our earlier work using CCD detection of the Er^3+^ emission in the work horse LIR thermometry material NaYF_4_:Er^3+^ demonstrated that Poisson noise was the main source determining the precision of temperature measurements. Other issues may the a slightly imperfect correction factor file for the detection system. One can also reduce the role of influence or need for spectral correction by measuring the AS/S ratio at a single known temperature T_0_ to determine a pre-factor, just as has been done for the ‘primary’ LIR thermometers discussed in Ref. (*44*). It is also evident that also for KSF:Mn^4+^ the peak broadening at elevated temperatures causes overlap between the vibronic emission lines. This can also explain the larger deviations at higher temperatures. The role of reabsorption at higher Mn^4+^ concentrations can also affect the performance. In the field of luminescence thermometry there has been a strong focus on finding materials with high relative sensitivity *S*_R_ to enable high theoretical precision. The challenges how to realize this and which effects and phenomena comprise the performance, also in actual applications, deserve more attention to come to reliable commercial applications of luminescence thermometers.

For the future and general applicability of luminescence thermometry it is important that more and superior calibration-free primary luminescence thermometry systems will be developed. Characteristics of promising AS/S vibronic LIR primary thermometers are stable hosts with luminescent ions showing strong and narrow well-resolved vibronic lines at different energies, a high luminescence quenching temperature and no change in vibrational energy with temperature

## MATERIALS AND METHODS

The synthesis of Mg_14_Ge_5_O_24_:Mn^4+^ was done following a previously reported method, employing the high-temperature solid-state reaction technique. GeO_2_ (99.999%), MgO (A.R.), and Mn_2_CO_3_ (A.R.) were used as raw materials without further purification. The raw materials were weighed according to the stoichiometric ratio, combined in a mortar, and thoroughly ground with 2 ml of anhydrous ethanol for 15 minutes. The mixture was then heated in a high-temperature tube furnace at 1350°C for 4 hours after ethanol evaporation. After cooling down to room temperature, the sample was retrieved, ground, and prepared for subsequent characterization. A series of MGO:x%Mn^4+^ samples with varying Mn^4+^ doping concentrations (0 ≤ x ≤ 3) were thus prepared. The sample of K_2_SiF_6_:0.1%Mn^4+^ was kindly provided by Current Chemicals.

Measurement and characterization: The x-ray powder diffraction patterns of MGO:xMn^4+^ phosphors were obtained using a Bruker D2 PHASER x-ray diffractometer (2θ = 10°-80°) with Cu Kα radiation (λ = 1.5418 Å, voltage = 30 kV). The crystal structure schematic diagram was generated using the VESTA program. Photoluminescence emission (PL) and excitation (PLE) spectra were collected using a Fluoro-3 fluorescence spectrometer equipped with a 450 W Xenon lamp excitation source. The temperature-dependent spectra and thermal cycle temperature-dependent spectra were measured using the Fluoro-3 fluorescence spectrometer from Edinburgh Instruments and the HCP621G-CUV1 heating stage from INSTEC, USA.
