# Supplementary material for: Exploring calibration-free luminescence thermometry with Mn4+ anti-Stokes/Stokes emission
Source: Sci Adv. 2026 Oct 16;12(42):eaeh4432. doi: 10.1126/sciadv.aeh4432 (PMC13674498; doi:10.1126/sciadv.aeh4432)
Supplement: Supplementary file 1 [file sciadv.aeh4432_sm.pdf]

Science Advances
AAAS

Supplementary Materials for

Exploring calibration-free luminescence thermometry with Mn^{4+} anti-Stokes/ Stokes emission

Guitong Zhang *et al.*

Corresponding author: Yuhua Wang, wyh@lzu.edu.cn; Andries Meijerink, a.meijerink@uu.nl

Sci. Adv. **12**, eaeh4432 (2026)
DOI: 10.1126/sciadv.aeh4432

This PDF file includes:

Figs. S1 to S11
Table S1

Figure S1(A) displays the XRD patterns of MGO phosphors with varying Mn^{4+} doping concentrations, indexed to the standard card (PDF # 04-010-1371) and shows that single phase materials are obtained without impurity phases such as $Mg_2(GeO_4)$ and $MgGeO_3$. Figure S1(B) shows the crystal structure of MGO using the VESTA program. Three different Ge^{4+} polyhedrons are formed in the Orthorhombic system (*36, 47*). Two types of four-coordinated GeO_4 are located on the (001) and (002) planes respectively, while the six-coordinated GeO_6 is situated on the (001) crystal plane. Since the ionic radius of Mn^{4+} is identical to that of Ge^{4+} (~0.54 nm) (*30*). and ions with d^3 configuration have a strong preference for octahedral coordination, Mn^{4+} will replace the Ge^{4+} site on the MnO_6 site without significant change in the crystal structure and no shift in peak position is observed with the increasing doping concentration in the XRD pattern.

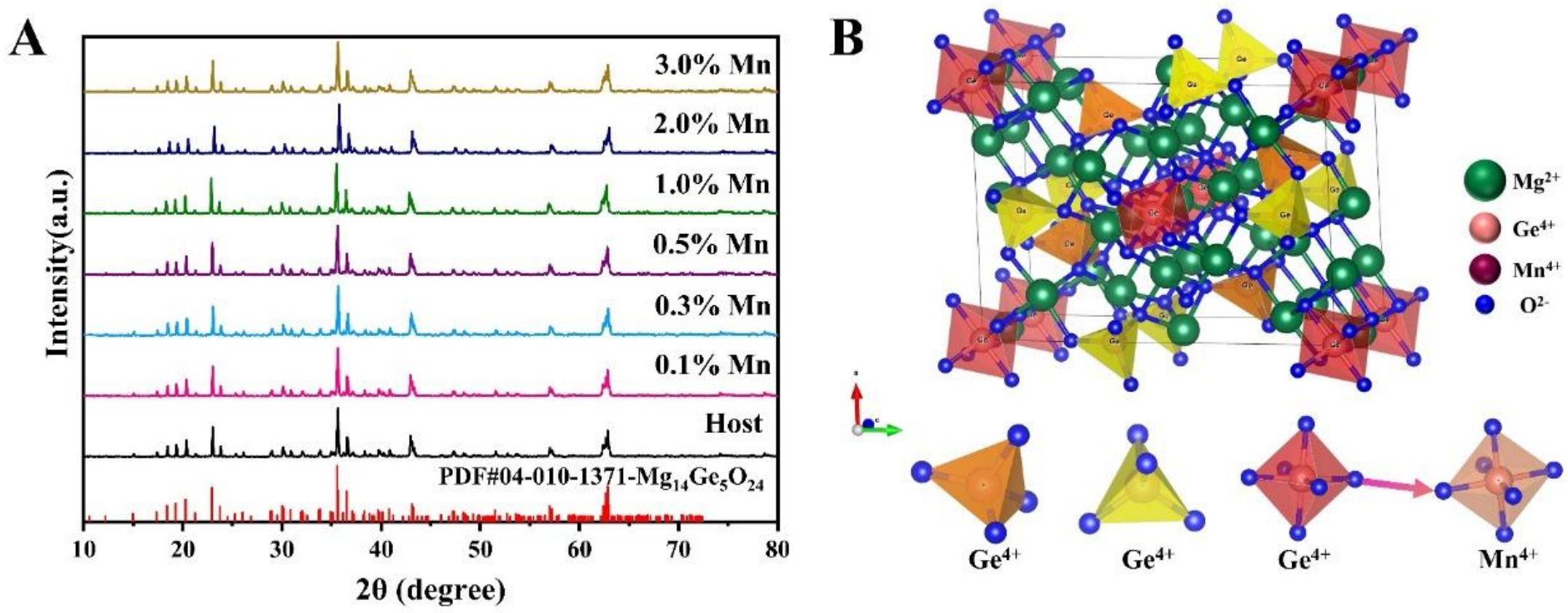

Figure S1. MGO:Mn^{4+} Material Characterization. (A) XRD patterns of $Mg_{14}Ge_5O_{24}$:x% Mn^{4+} (x=0,0.1,0.3,0.5,1,2,3) and the PDF standard card of $Mg_{14}Ge_5O_{24}$ as a reference. (B) Crystal structure of $Mg_{14}Ge_5O_{24}$, with four-coordinated GeO_4 and six-coordinated GeO_6 structures in different environments.

SEM images and particle size statistical analysis of MGO (Figures S2(A, B)) indicate that the prepared particles have a wide size distribution and there is agglomeration after sintering. Particle sizes range from approximately 1 micron to 6 microns, with an average size of 2.6 microns. Elemental mapping (Fig. S2(C)) illustrates uniform distribution within 5-micron agglomerated particles. Further analysis via TEM and HRTEM (Fig. 2(D) and (E)) shows irregular particle shapes and non-uniform sizes. HRTEM indicates a measured crystal plane spacing of 0.372 nm, corresponding to the (121) spacing of 0.375 nm in the standard card.

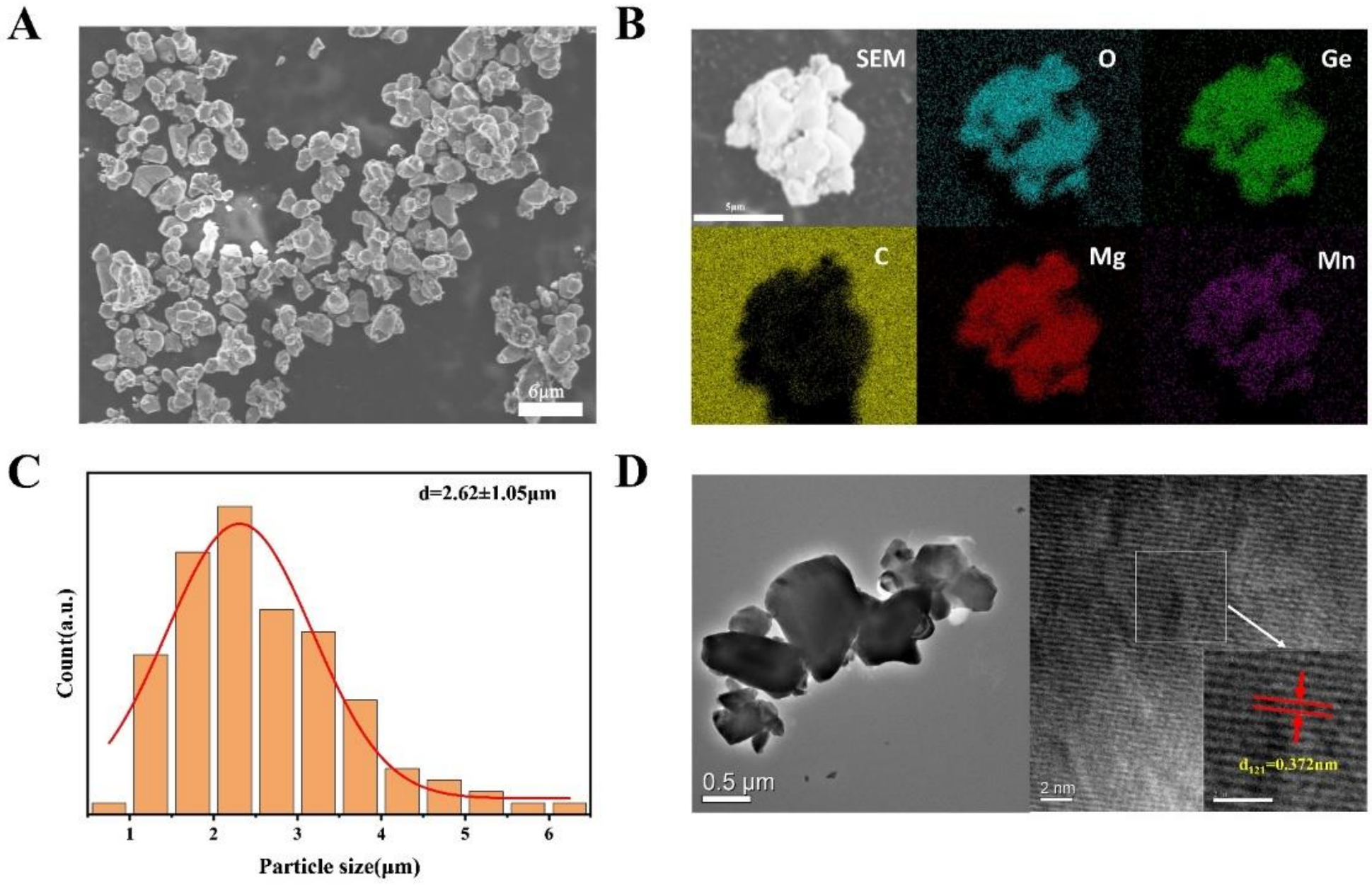

Figure S2. Morphological Features and Element Distribution of MGO:1%Mn^{4+}
(A) SEM image of $Mg_{14}Ge_5O_{24}$:1% Mn^{4+}, (B) Elemental mapping of $Mg_{14}Ge_5O_{24}$:1% Mn^{4+}. (C) Particle size distribution of $Mg_{14}Ge_5O_{24}$:1% Mn^{4+} determined from analysis of the SEM image (D) TEM image and HRTEM image of $Mg_{14}Ge_5O_{24}$:1% Mn^{4+}

To optimize the Mn^{4+} doping concentration in MGO, the emission spectra of different doping concentrations are measured as shown in Fig. S3(A). With the increase of manganese ion concentration, the luminescence intensity increases up to 1% and then the intensity decreases continuously due to the concentration quenching. Fig. S3(B) shows that the internal quantum efficiency of $Mg_{14}Ge_5O_{24}$:1% Mn^{4+} is around 60% and the external quantum efficiency 24% by the integrating sphere method under 420nm excitation (*48-49*).

The luminescence lifetimes of MGO with different Mn^{4+} doping concentrations were calculated by single exponential fitting of the luminescence decay curves recorded for the Mn^{4+} emission under pulsed nm excitation, Figs. S3(C) and S3(D). With the increase of the doping concentration, the average distance between the Mn^{4+} ions decreases allowing for energy migration to quenching centers resulting in a drop of the average decay time from 3 ms for 0.1%Mn^{4+} to 1.4 ms for 3%Mn^{4+} 1.4ms (*50-51*).

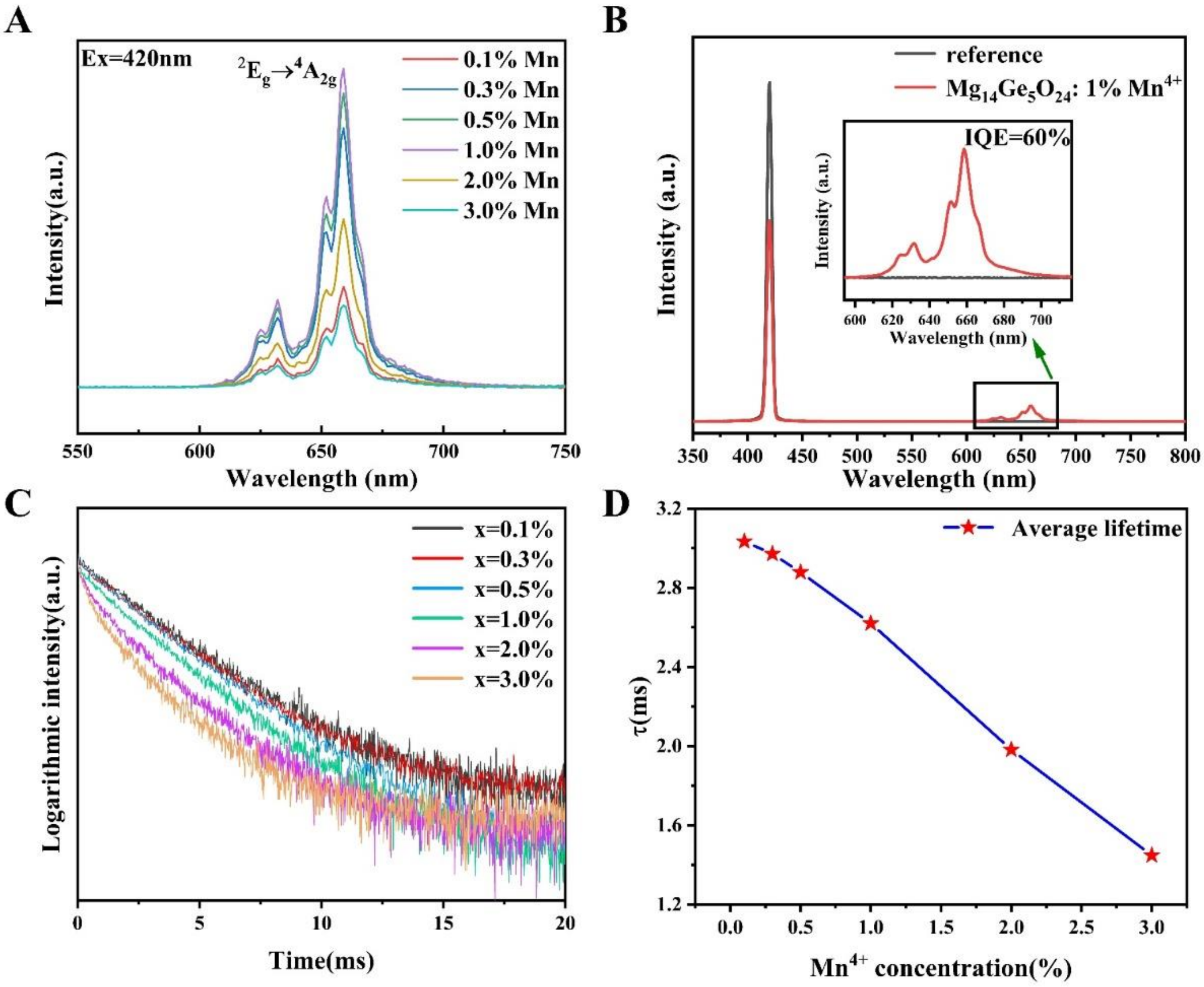

Figure S3. Optical properties of Mn^{4+} concentration-doped materials (A) Concentration-dependent emission spectra of $Mg_{14}Ge_5O_{24}$: x% Mn^{4+} under the 420 nm excitation (x=0.1, 0.3, 0.5,1, 2, 3). (B) Quantum efficiency measurement of the $Mg_{14}Ge_5O_{24}$:1% Mn^{4+} using an integrating sphere (C) Luminescence decay curves of $Mg_{14}Ge_5O_{24}$:x% Mn^{4+} at room temperature for x=0.1, 0.3, 0.5, 1, 2, 3 (λ_{exc}=420nm, λ_{em}=660nm) (D) Average lifetime with changing Mn^{4+} concentration as determined from single exponential fits for the decay curves in (C).

To evaluate the temperature dependence of the I_{AS}/I_S ratio in MGO:1%Mn^4 emission spectra were recorded under identical experimental conditions while raising the temperature. Examples of emission spectra are shown in Fig. 2 of the main text. After correction of the spectra for the instrumental response and conversion to an energy scale, the ratio of the peak intensities was determined and plotted in Fig. 3 of the main text. These ratios were determined from the temperature dependent intensities of both the anti-Stokes (AS) and Stokes (S) vibronics. In Figure S4 the intensities for the ν_6, ν_4 and total integrated vibronic intensity ν_{tot} are plotted as function of temperature. For ν_6 and ν_4 the peak intensity (photon flux per constant energy interval (ΦE) in the converted spectrum) is plotted for the AS and S peaks (Figs. S3(A,B)) while for ν_{tot} the intensity was integrated over the full anti-Stokes or Stokes vibronic spectral region (Fig.

S3(C)). It can be observed that the intensities of AS vibronics increases at the expense of the S vibronics. The total intensity remains approximately constant, consistent with the high thermal quenching temperature of the Mn^{4+} luminescence in MGO. In Fig. S3(D) the Boltzmann fit to the $\ln(\nu_{tot}'/\nu_{tot})$ shows that a good fit is obtained for an effective vibrational energy of 271 cm^{-1}. This fit serves as calibration curve for temperature sensing with MGO:Mn^{4+}.

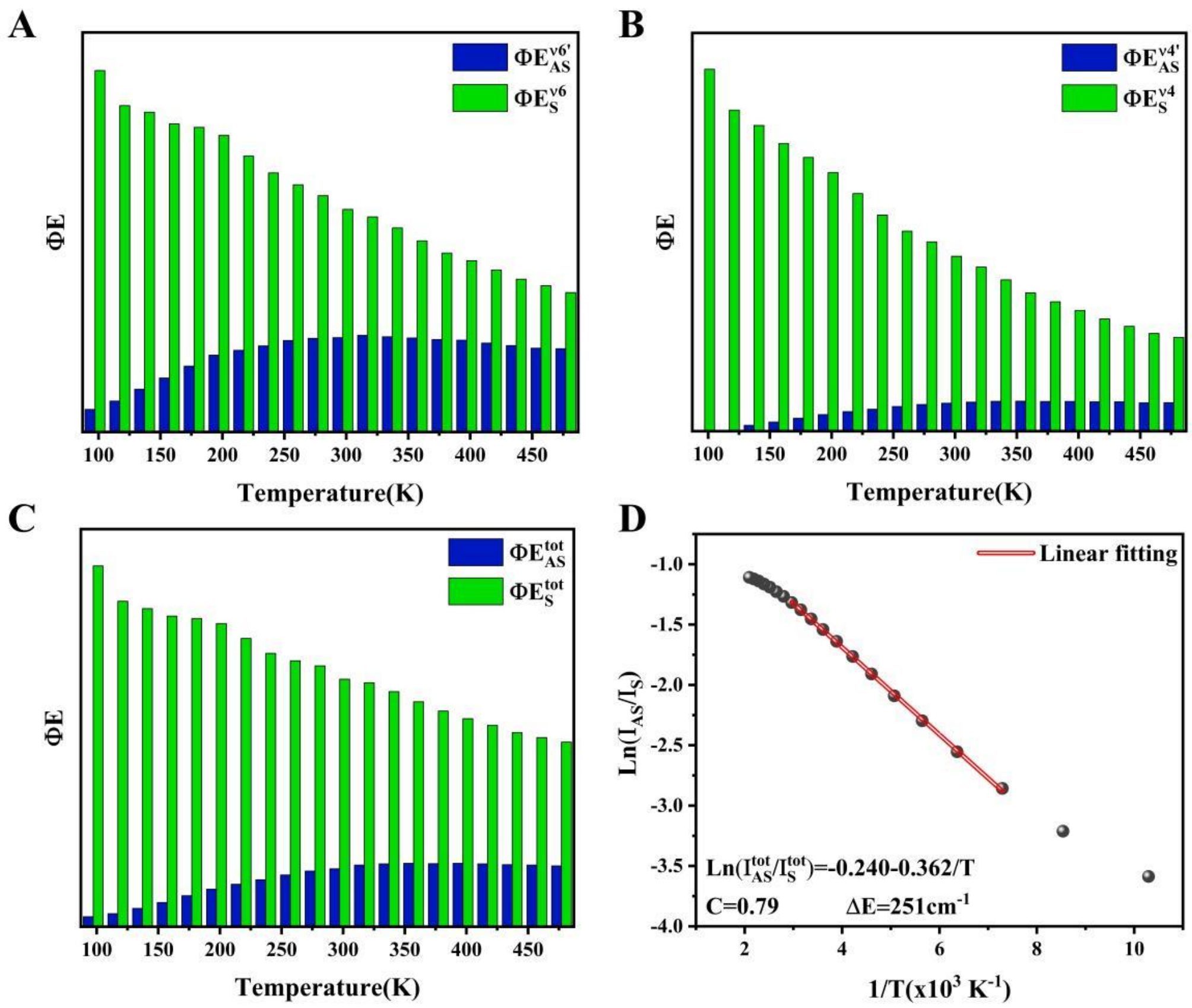

Figure S4. Temperature dependence MGO:Mn^{4+} Temperature dependence of emission intensities of anti-Stokes (ΦE_{AS}) and Stokes (ΦE_S) emission as determined from emission spectra after correction for the instrumental response and converting the spectra to a photon flux per constant energy interval (ΦE) vs. energy. The peak intensities for (A) ν_6 (Stokes) and ν_6' (anti-Stokes) and (B) ν_4 and ν_4' are shown. (C) For total Stokes and anti-Stokes vibronic intensities, ΦE_{tot}, the integrated intensities are plotted. The ratios of $\Phi E_{AS}/\Phi E_S$ (or I_{AS}/I_S) determined from these data are plotted in Fig. 3 of the main text and Fig. S5. (D) Experimentally measure intensity ratio of anti-Stokes/Stokes vibronics for ν_{tot}/ν_{tot}'. The red line is a Boltzmann fit to Eq. (6). Fitting parameters for the best fit are indicated.

To test the temperature sensing performance of MGO:Mn^{4+} the absolute sensitivities (S_A) and relative sensitivity (S_R) were determined from Figs. 3(C) and 3(D) for ν_6 and ν_4 and from Fig. S4(D) for ν_{tot}. In Fig. S5 the temperature dependence of S_A is shown. The relative sensitivities S_R for ν_6 and ν_4 are included in Fig. 5 of the main text and for ν_{tot} in Fig. S5(D).

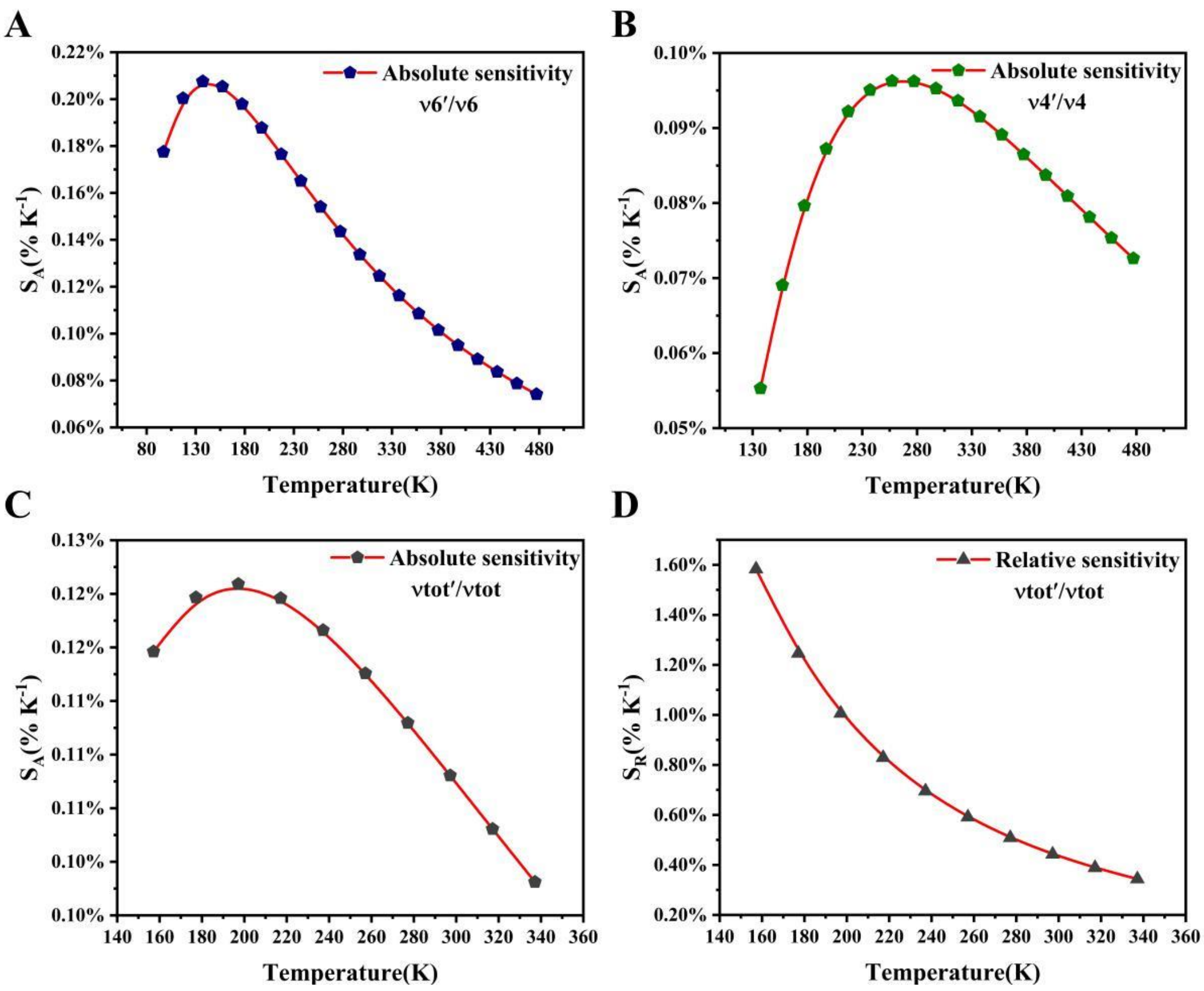

Figure S5. Absolute sensitivity and relative sensitivity of MGO:Mn^{4+} (A,B,C) Absolute sensitivity S_A values for the AS/S vibronic intensity for the ν_6, ν_4 ,and ν_{tot} vibrational modes in MGO:1%Mn^{4+} according to the fits Fig. 3 (C, D) and SI Fig. S4 (D). (D) Relative sensitivity S_R values for the AS/S vibronic intensity for the ν_{tot} vibrational modes in MGO:1%Mn^{4+} according to SI Fig. S4 (D)

To further evaluate the reliability and reproducibility for temperature sensing thermal cycling experiments were carried out as shown in Fig. 5 (C) and (D). Uncertainties in temperature were calculated from the variations in temperature for the same setpoint and are included in Table S1. Uncertainties around 1 K are obtained below and around room temperature. For the high temperature regime where S_R drops the uncertainty increases.

TABLE S1 Temperature uncertainty of MGO:1%Mn^{4+} at different temperatures

Temperature uncertainties calculated at different temperatures from the thermal cycling experiments shown in Fig.5 of the main text and determination of temperatures from the intensity ratios ν_6'/ν_6 and ν_4'/ν_4 for MGO:1%Mn^{4+}.

Vibrations	Temperature	97K	173K	273K	373K	473K
ν6-ν6′	δT(K)	0.73	2.1	1.8	5.2	8.7
Vibrations	Temperature	137K	173K	273K	373K	473K
ν4-ν4′	δT(K)	0.48	1.4	1.1	2.1	7.0

In the main text two examples of experimentally measured emission spectra and fits to a sum of Gaussion was shown. For completeness, Figure S6 shows fits for other temperatures and also the temperature dependence of peak positions and integrated intensities for vibronic lines.

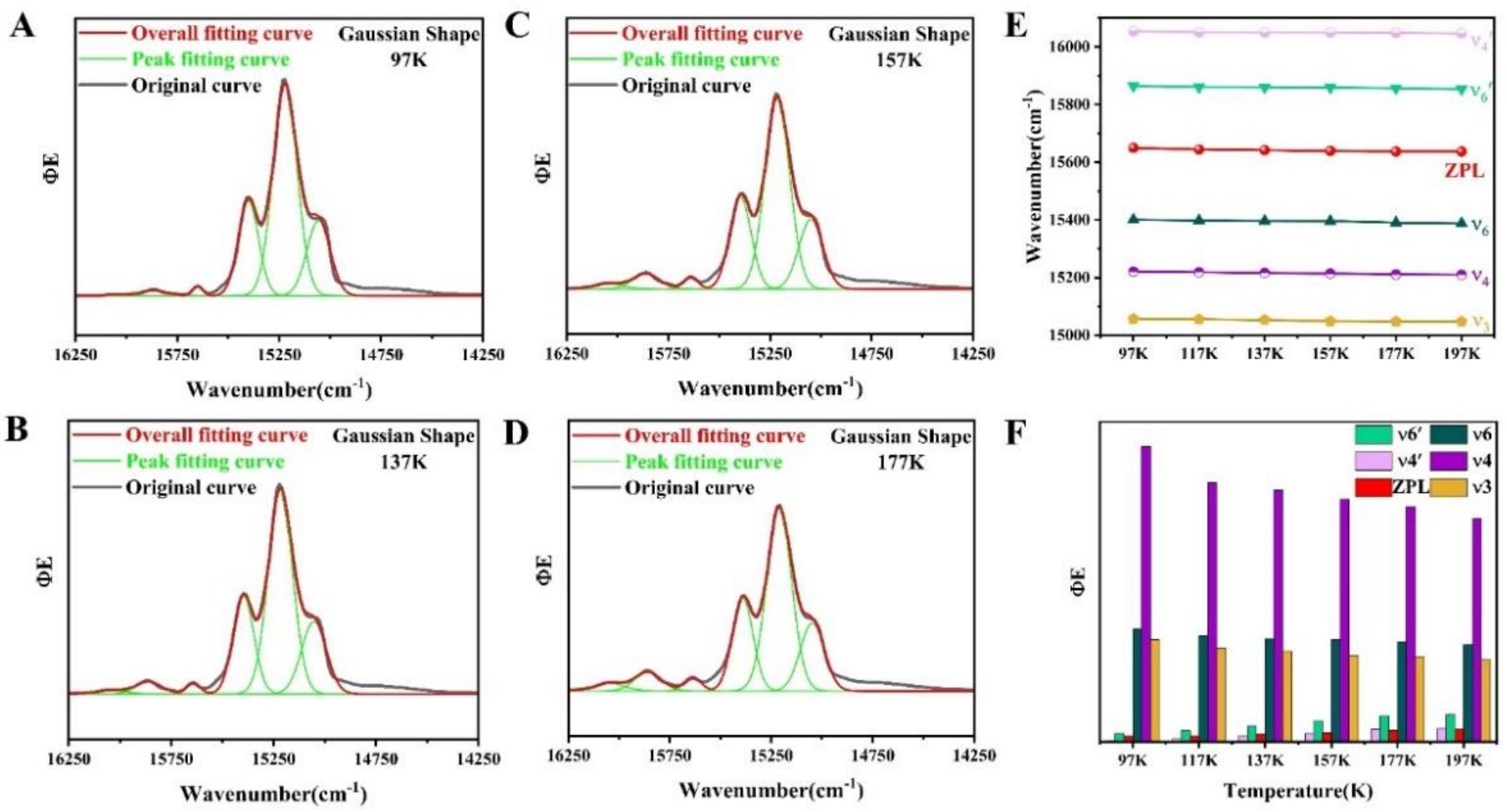

Figure S6. Temperature-dependent behavior under Gaussian fitting(A-D) Emission spectra of MGO:Mn^{4+} at four temperatures fitted to a sum of Gaussians to extract the intensities of individual Stokes and anti-Stokes vibronic lines. (E-F)The peak position and intensity of each emission peak vary with temperature (97K-197K).

From the results it is evident that fitting to a sum of Gaussian does not results in AS/S intensity ratios that match the theoretically expected ratios. For completeness, also fitting to a sum of Lorentzians was done. In Figures S7 and S8 fitted curves are shown. Again, reasonably good fits for the experimentally measured is obtained.

However, the intensity ratios deviate strongly from the theoretically expected ratios as shown in Figs. S7(C) and (E). Good fits to the Boltzmann Eq. (6) can be obtained as shown in Figs. S7 (D) and (F) but the vibrational energies deviate from the actual for ν_6 and ν_4.

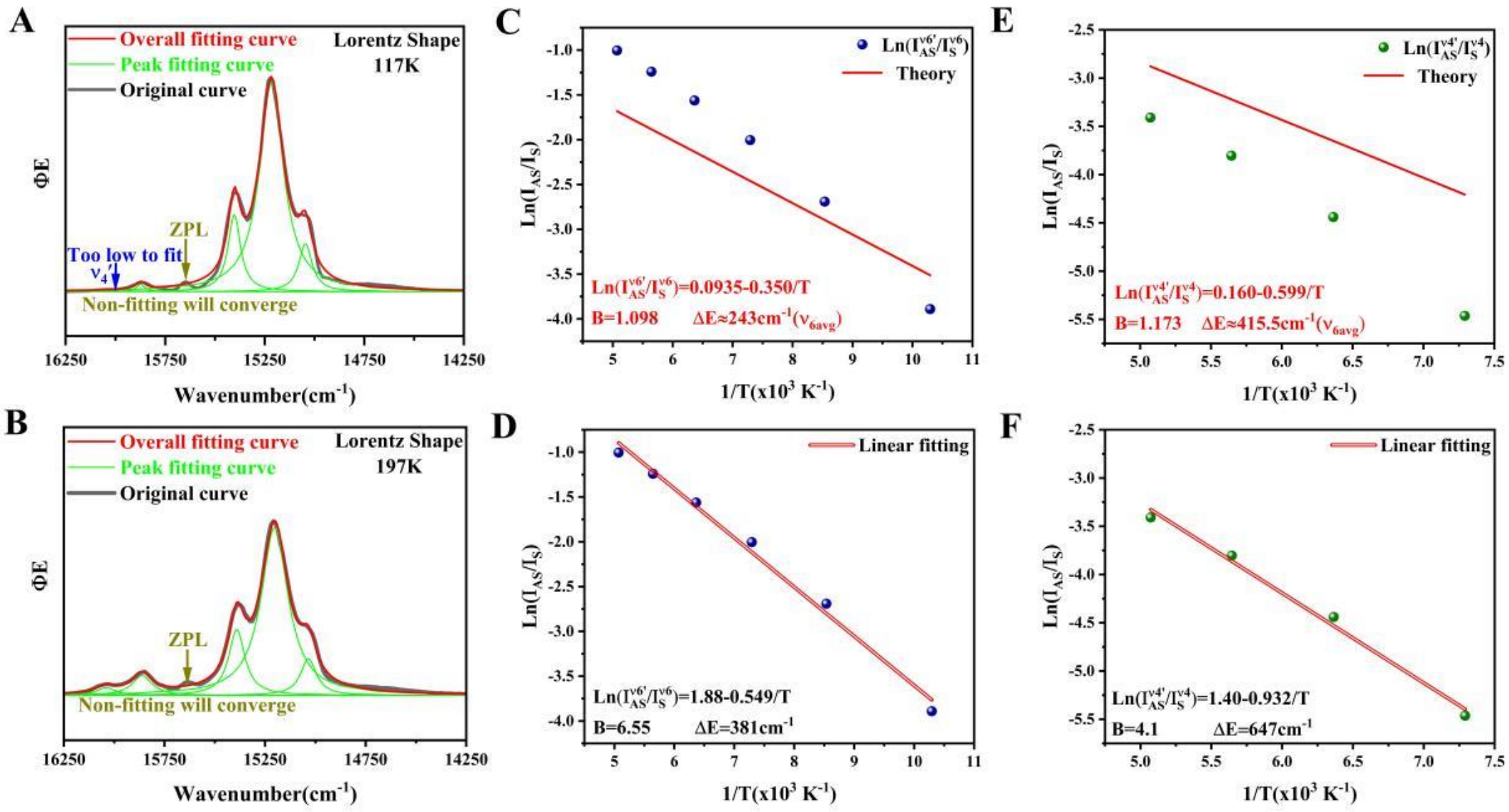

Figure S7. Temperature-dependent behavior under Lorentz fitting (A, B) Examples of emission spectra of MGO:Mn^{4+} at 117K and 197K fitted to a sum of Lorentzians to extract the intensities of individual Stokes and anti-Stokes vibronic lines. (C) Temperature dependence of the *LIR* for ν_6'/ν_6 and (E) for ν_4'/ν_4 determined from the Lorentz fits plotted together with the theoretical temperature dependence for intensity ratios. In (D) and (F) fits to Eq. (6) are depicted for the same data points. Fitting parameters are included in the Figures.

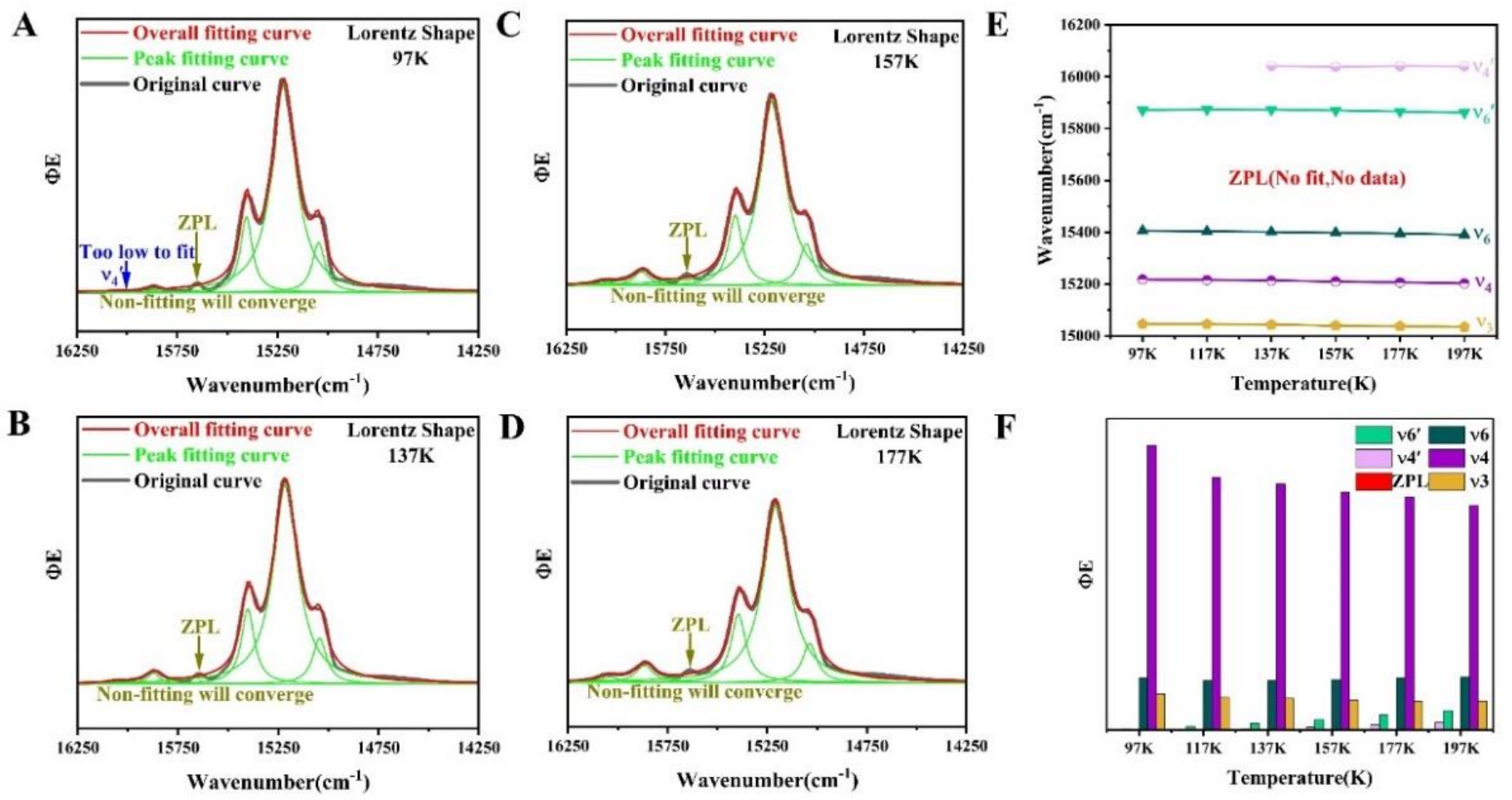

Figure S8. Temperature-dependent behavior under Lorentz fitting(A-D) Emission spectra of MGO:Mn^{4+} at six temperatures fitted to a sum of Lorentzians to extract the intensities of individual Stokes and anti-Stokes vibronic lines, (E, F) The peak position and intensity of each vibronic emission peak as determined from the Lorentz fits for different temperatures (97K-197K)

In addition to using the luminescence intensity ratio (LIR) for temperature measurement, the luminescence lifetime can also be used. Luminescence decay curves of MGO:1%Mn^{4+} between 77K and 773K are shown in Figures S9(A, B). Decay times are determined by single exponential curve fitting, as shown in Figure S9(C). With the increase in temperature, the lifetime decreases from 4.5 ms at 77K to 0.2 ms at 773K. Previously a complete model was reported to explain the variation of Mn^{4+} emission lifetime by (1) enhanced radiative probabilities for vibronic lines (scaling with the phonon occupation number n for anti-Stokes lines and (n+1) for Stokes lines) and (2) thermal quenching of the luminescence via the $^4T_2$ excited state at higher temperatures (*21*). For the vibronic enhancement the temperature dependence of the decay time is described by:

$$\tau_R(T) = \frac{\tau_R(0)}{\coth(\frac{E_{\mathrm{vib}}}{2k_BT})} \qquad \text{(S1)}$$

where $\tau_R(0)$ represents the radiative lifetime at 0K. In Fig. S9(C), a fit to Eq. S1 is been plotted for $\tau_R(0)$=4.64ms and E_{vib} =284cm^{-1} (the effective phonon energy) and accurately follows the experimentally measured Mn^{4+} emission lifetime up to ~400K, confirming that the decay of the ^2E state up to this temperature is mainly radiative. The luminescence lifetime above 400K drops off faster than predicted by Eq. (S1) indicating

that thermally activated non-radiative transitions start to contribute to the decay of the ^2E state. The luminescence quenching temperature can be obtained from the temperature where the emission lifetime is reduced to half of the radiative lifetime. The green line is obtained by dividing the radiative lifetime by 2 and the crossing point between this line and the experimental data is about 700K and represent the quenching temperature. This value is very close to the previously reported value of 720K (*21*).

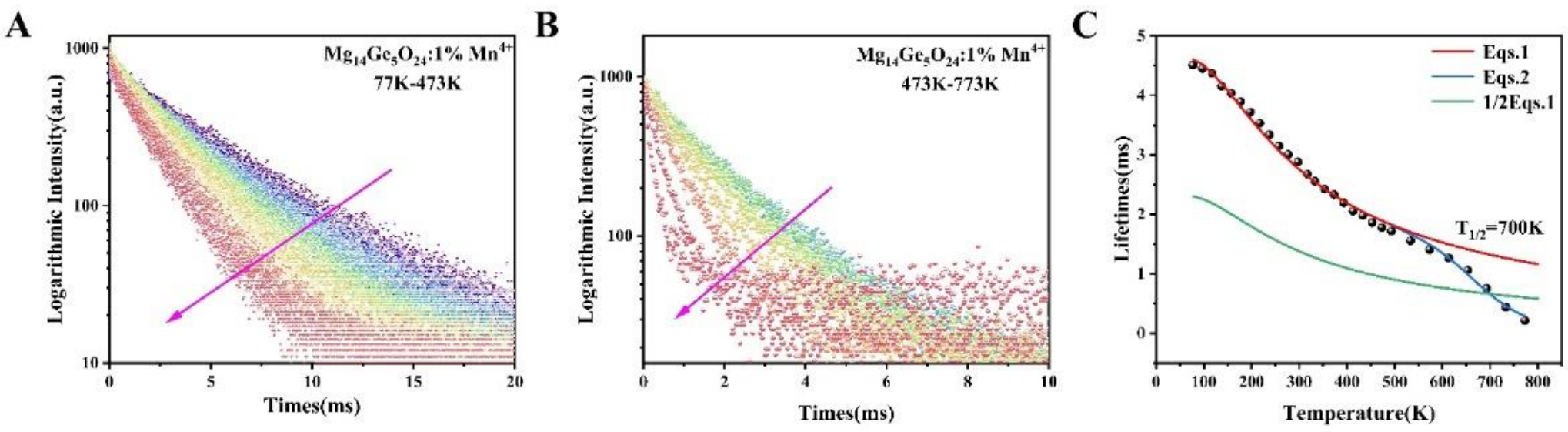

Figure S9. Temperature dependence of decay time (A) at low temperatures (77K-473K) and (B) at high temperatures (473K-773K), (C) Temperature dependence of the Mn^{4+} emission lifetime for $Mg_{14}Ge_5O_{24}$:1% Mn^{4+}. The red and blue lines represent fits to Eqs. (S1) and (S2), respectively. The green line gives the fit for Eq. (S1) (red line) divided by 2.

For a fit of the full temperature region the influence of thermally activated luminescence quenching can be included in Eq.(S1) and gives (*52*):

$$\tau(T) = \frac{\tau_R(T)}{1+\left(\frac{\tau_R(T)}{\tau_{NR}}\right)exp(-\frac{\Delta E}{k_B T})} \quad \text{(S2)}$$

where $\frac{1}{\tau_{NR}}$ is the non-radiative decay rate ($\frac{1}{\tau_{NR}} = 3.6 \times 10^{12}\mathrm{s}^{-1}$) and $\tau_R(T)$ is the radiative lifetime as described by Eq.(S1). A good fit to Eq. (S2), given by the blue line, is obtained for an activation energy for thermal quenching ΔE of 6344 cm^{-1} (*53-54*).

Just as for MGO:Mn^{4+} the temperature dependence of the I_{AS}/I_S ratio in KSF:0.1%Mn^{4+} was determined by recording emission spectra under identical experimental conditions while raising the temperature under 450 nm excitation. Examples of emission spectra are shown in Fig. 6 of the main text. After correction of the spectra for the instrumental response and conversion to an energy scale, the ratio of the peak intensities was determined and plotted in Fig. 7 of the main text. These ratios were determined from the temperature dependent intensities of both the anti-Stokes (AS)

and Stokes (S) vibronics. In Figure S10 the intensities for the ν_6, ν_4, ν_3 and total integrated vibronic intensity ν_{tot} are plotted as function of temperature. For ν_6, ν_4 and ν_3 the peak intensity (photon flux per constant energy interval (ΦE) in the converted spectrum) is plotted for the AS and S peaks (Figs. S10(A,B,C)) while for ν_{tot} the intensity was integrated over the full anti-Stokes or Stokes vibronic spectral region (Fig. S10(D)). It can be observed that initially both the intensities of the S and AS vibronics increases with temperature although the AS intensities increase faster, giving rise to the increasing AS/S ratio as expected. The initial increase of the total intensity can be explained by stronger absorption at 450 nm due to vibrationally induced absorption that increases with the phonon occupation number of odd parity vibrations that enable the parity forbidden $^4A_2 \rightarrow ^4T_2$ transition in octahedral symmetry. The temperature dependence of ratios of the AS/S vibronics as shown in Fig. 7 of the main text were determined from data in Fig. S10.

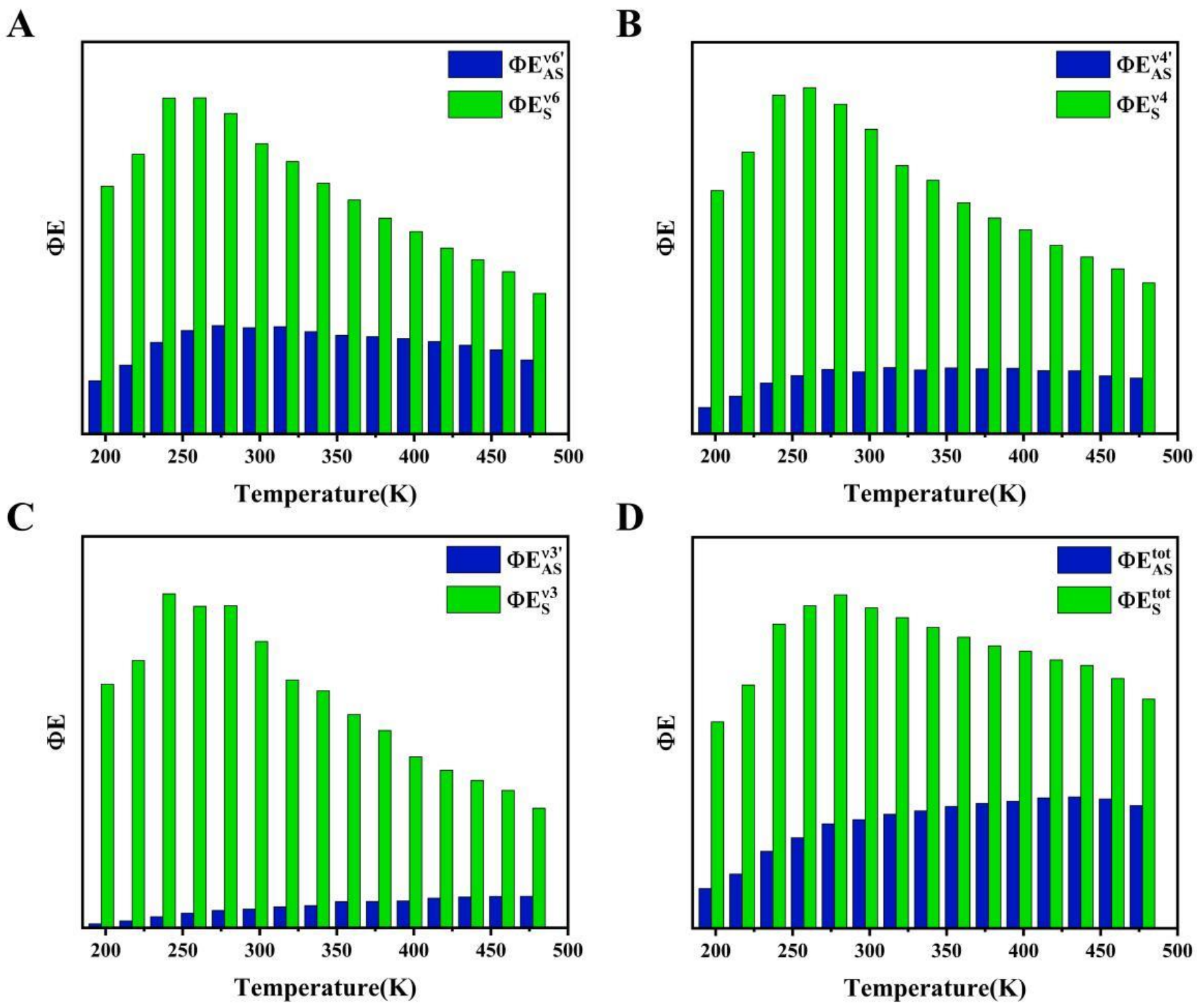

Figure S10. Temperature dependence of emission intensities of anti-Stokes (ΦE_{AS}) and Stokes (ΦE_S) emission of KSF:0.1%Mn^{4+} as determined from emission spectra after correction for the instrumental response and converting the spectra to a photon flux per constant energy interval (ΦE) vs. energy. The peak intensities for (A) ν_6 (Stokes) and ν_6' (anti-Stokes), (B) ν_4 and ν_4' and (C) ν_3 and ν_3' are shown. (D) For total Stokes and anti-Stokes vibronic intensities, ΦE_{tot}, the integrated intensities are plotted. The ratios of $\Phi E_{AS}/\Phi E_S$ (or I_{AS}/I_S) determined from these data are plotted in Fig. 7 of the main text.

For evaluation of the performance as a luminescence thermometer the absolute and relative sensitivities of the *LIR* for the different vibrational modes were determined as a function of temperature. In the main text the more important relative sensitivities S_R are shown in Fig. 8. For completeness, it is also good to have insight in the absolute sensitivities S_A. Figure S11 shows S_A for the different vibrational modes as calculated using Eq. (7). For the higher energy modes S_A is small at low temperatures where the vibrational modes are not yet being occupied. The relative sensitivity S_R is however high as this is determined by dividing by the small value of S_A. For the higher energy ν_4 and ν_3 vibrations S_A reaches a maximum at ~200K and ~450K.

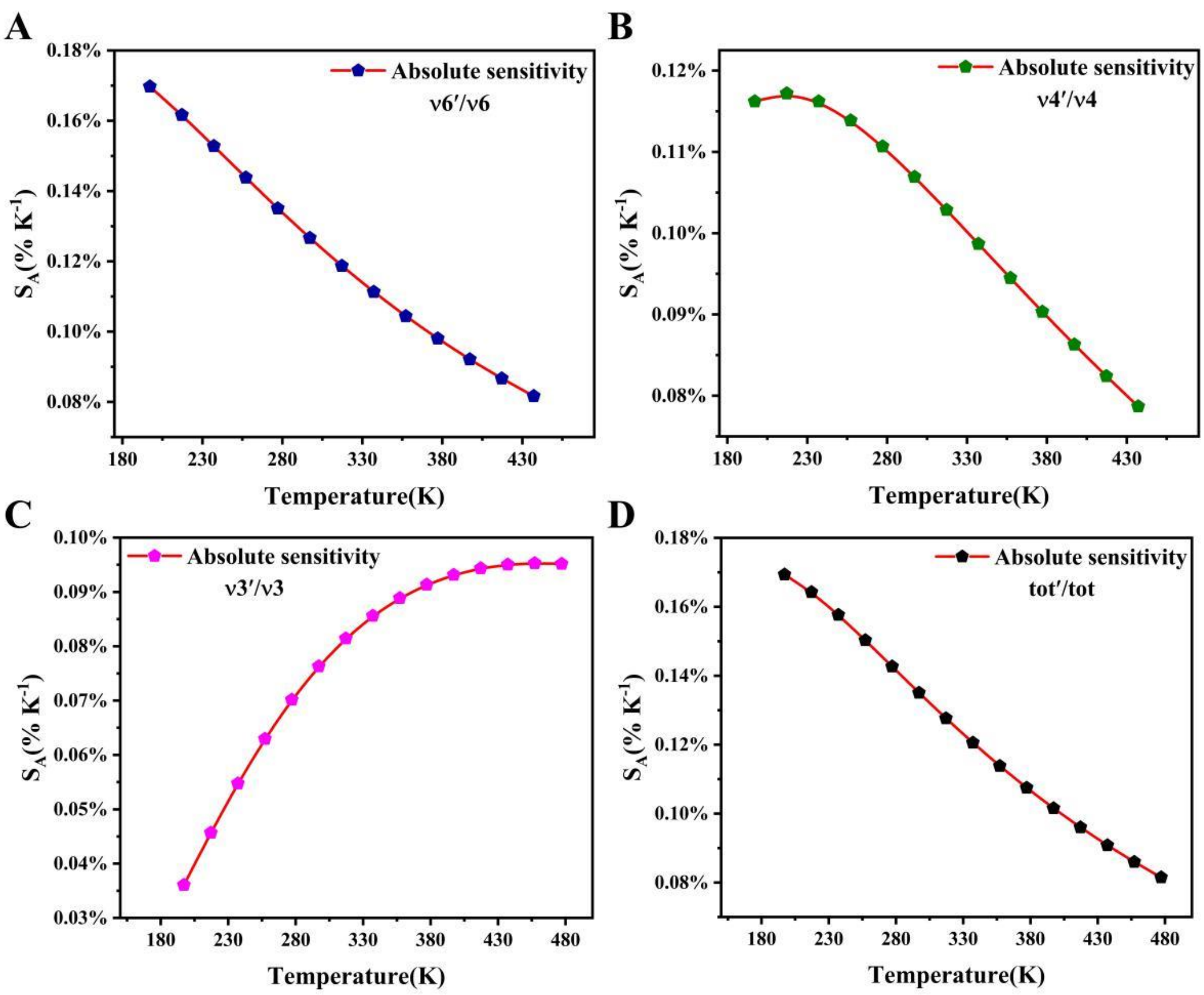

Figure S11. Absolute sensitivity of KSF:Mn^{4+}.Based on the fitting results shown in Figures 7, the absolute sensitivity S_A values of the MGO:1%Mn^{4+} for the vibrational modes of ν_6 (A), ν_4(B), ν_3(C) and ν_{tot}(D) were calculated.
